# Supplementary material for: Functional diversification across yeast lineages of the nucleus-vacuole junction forming protein Nvj1
Source: bioRxiv. 2026 Sep 10:2026.09.05.749613. Preprint. [Version 1] doi: 10.64898/2026.09.05.749613 (PMC13622503; doi:10.64898/2026.09.05.749613)
Supplement: Supplementary file 1 [file media-1.pdf]

Kramer et al.

A

Group 6	*Z. gambellarensis*	*Z. pseudorouxii*	*Z. mellis*	*Z. siamensis*	*Z. kombuchaensis*	*Z. lentus*	*Z. bisporus*	*Z. bailii*	*Z. florentina*	*Z. chibaensis*	*Z. mrakii*	*Z. danielsina*	*H. aurorensis*
Z. gambellarensis	-												
Z. pseudorouxii	72.1	-											
Z. mellis	65.7	74.5	-										
Z. siamensis	64.3	75.2	93.9	-									
Z. kombuchaensis	57.4	59.3	55.8	57.7	-								
Z. lentus	56.8	57.7	54.1	56.0	83.6	-							
Z. bisporus	59.7	57.8	53.9	53.9	70.6	68.6	-						
Z. bailii	57.9	54.6	54.8	53.7	71.3	66.8	72.8	-					
Z. florentina	31.6	32.1	30.5	30.8	30.4	30.5	29.3	29.9	-				
Z. chibaensis	31.2	31.1	28.3	28.3	28.0	28.9	30.4	30.7	73.4	-			
Z. mrakii	29.0	27.8	27.5	26.5	28.8	30.1	29.3	30.4	36.9	36.6	-		
Z. danielsina	29.9	29.1	29.9	28.8	31.9	31.6	33.6	32.1	40.3	40.9	47.4	-	
H. aurorensis	24.7	22.9	24.7	24.7	27.7	27.1	25.8	27.4	20.7	22.0	21.7	21.6	-

Group 7	*L. mirantina*	*L. kluyveri*	*L. meyersii*	*L. fantastica*	*L. lanzarotensis*	*L. nothofagi*	*L. dasiensis*	*L. fermentati*	*L. waltii*	*L. quebecensis*	*L. thermotolerans*
L. mirantina	-										
L. kluyveri	27.4	-									
L. meyersii	25.5	33.1	-								
L. fantastica	29.4	31.4	47.0	-							
L. lanzarotensis	25.8	29.1	47.5	66.8	-						
L. nothofagi	28.2	33.0	41.4	37.6	38.2	-					
L. dasiensis	25.4	28.2	31.8	32.0	32.4	38.7	-				
L. fermentative	32.9	32.9	29.7	27.2	27.3	29.2	26.9	-			
L. wellie	27.5	30.9	32.3	32.6	33.1	33.0	30.7	30.9	-		
L. quickness	27.1	33.5	35.8	35.4	37.4	38.6	35.1	36.7	41.9	-	
L. thermotolerance	30.5	34.5	36.8	34.9	36.9	37.7	36.8	34.8	42.9	76.5	-

Group 9	*H. vinae*	*H. valbyensis*	*H. pseudoguilliermondi*	*H. uvarum*	*H. clermontiae*
H. vinae	-				
H. valbyensis	17.4	-			
H. pseudoguilliermondi	23.7	27.5	-		
H. uvarum	24.3	27.2	60.5	-	
H. clermontiae	21.8	27.8	59.4	67.5	-

Group 8	*E. gossypii*	*A. aceri*	*K. nonfermentans*	*K. aestuarii*	*K. siamensis*	*K. wickerhamii*	*K. lactis*	*K. dobzhanskii*	*K. starmeri*	*E. coryli*	*E. cymbalariae*	*E. sinecaudum*
E. gossypii	-											
A. aceri	48.0	-										
K. nonfermentans	13.7	16.0	-									
K. aestuarii	17.0	19.3	45.6	-								
K. siamensis	15.7	17.4	46.2	77.6	-							
K. wickerhamii	13.1	14.1	28.5	28.6	27.3	-						
K. lactis	15.8	17.7	25.3	25.5	26.2	29.6	-					
K. dobzhanskii	16.4	17.7	29.4	30.0	27.9	31.0	53.4	-				
K. starmeri	15.2	18.9	22.4	28.5	27.5	27.8	34.7	35.1	-			
E. coryli	15.7	20.9	17.4	19.4	18.2	18.6	19.6	22.5	20.0	-		
E. cymbalariae	18.1	18.7	24.4	26.3	24.8	20.9	20.9	20.2	21.2	24.6	-	
E. sinecaudum	13.7	15.6	20.8	21.6	22.0	19.9	19.4	22.0	22.4	25.7	28.4	-

Group 10	*W. chambardii*	*W. piperi*	*W. mucosus*	*W. hampshirensis*	*C. fabianii*	*C. jadinii*	*C. suaveolens*	*C. saturnus*	*C. misumaiensis*	*C. maclurae*	*W. ciferrii*	*W. alni*	*W. canadensis*
W. chambardii													
W. piperi	15.7	-											
W. mucosus	18.2	24.4	-										
W. hampshirensis	18.4	20.5	26.0	-									
C. fabianii	21.1	24.3	26.5	36.1	-								
C. jadinii	19.8	18.5	19.2	32.2	31.3	-							
C. suaveolens	17.9	22.8	26.8	34.9	35.1	38.2	-						
C. saturnus	18.9	22.4	26.8	35.9	32.8	38.5	86.3	-					
C. misumaiensis	18.9	18.4	21.7	36.4	38.9	33.7	40.8	38.6	-				
C. maclurae	20.8	23.2	28.1	42.1	43.3	38.8	43.7	42.7	73.8	-			
W. ciferrii	17.1	17.6	21.2	26.5	26.8	24.6	31.4	30.7	23.9	30.8	-		
W. alni	19.3	21.5	27.7	32.4	31.9	28.0	33.7	33.7	30.7	33.5	29.7	-	
W. canadensis	22.2	24.6	31.4	37.0	34.1	34.5	39.5	38.1	34.7	37.2	35.2	47.7	-

Group 11	*K. floccosa*	*K. populi*	*Komatagataella*	*K. pastoris*	*K. phaffi*	*K. kurtzmanii*
K. floccoa	-					
K. populi	25.0	-				
Komatagataella	23.9	73.4	-			
K. pastoris	23.0	72.7	72.7	-		
K. phaffi	23.0	71.8	71.8	90.2	-	
K. kurtzmanii	23.0	71.8	71.8	90.2	98.7	-

B

Interclade	*K. pastoris*	*H. pseudoguilliermondi*	*C. suaveolens*	*K. wickerhamii*	*V. tropicalis*	*L. quebecensis*	*N. baii*	*T. maleeae*	*S. cerevisiae*	*N. bracarensis*	*K. yasuniensis*
K. pastoris	-										
H. pseudoguilliermondi	23.2	-									
C. suaveolens	13.1	22.6	-								
K. wickerhamii	13.7	18.7	14.0	-							
V. tropicalis	17.0	11.9	14.5	13.2	-						
L. quebecensis	13.4	17.4	13.1	20.4	16.0	-					
N. baii	18.5	15.1	17.8	14.2	22.6	18.4	-				
T. maleeae	13.3	12.6	12.1	19.2	20.3	24.1	20.5	-			
S. cerevisiae	11.6	16.7	15.4	22.0	21.6	21.4	25.6	28.9	-		
N. bracarensis	12.2	19.1	17.0	15.5	17.1	18.2	20.8	22.8	24.6	-	
K. yasuniensis	15.8	18.0	17.0	16.0	21.0	18.0	21.1	17.8	22.2	32.1	-

Group 1	*S. uvarum*	*S. eubayanus*	*S. arboricola*	*S. kudriavzevii*	*S. jurei*	*S. mikatae*	*S. cerevisiae*	*S. paradoxus*
S. uvarum	-							
S. eubayanus	89.4	-						
S. arboricola	67.6	67.5	-					
S. kudriavzevii	68.6	68.8	71.1	-				
S. jurei	68.1	67.7	67.6	67.5	-			
S. mikatae	69.7	70.2	69.7	68.8	90.8	-		
S. cerevisiae	69.9	69.8	72.5	67.7	75.7	78.5	-	
S. paradoxus	73.4	72.4	74.1	72.5	81.0	83.2	86.9	-

Group 2	*N. bacillisporus*	*N. castellii*	*N. glabrata*	*N. uthaithanina*	*N. delphensis*	*N. bracarensis*	*N. kungkrabaensis*	*N. nivariensis*
N. bacillisporus	-							
N. castellii	26.9	-						
N. glabrata	18.2	27.5	-					
N. uthaithanina	27.6	23.9	44.7	-				
N. delphensis	22.2	28.9	45.6	55.9	-			
N. bracarensis	23.5	27.1	47.9	58.4	60.7	-		
N. kungkrabaensis	23.2	28.0	46.4	55.7	64.7	69.7	-	
N. nivariensis	23.2	28.0	45.9	57.0	64.7	70.6	97.4	-

Group 3	*K. viticola*	*K. bromeliacearum*	*K. sinensis*	*K. naganishii*	*K. taianensis*	*K. unispora*	*K. yasuniensis*	*K. servazzi*	*K. solicola*	*K. aerobia*	*K. aquatica*	*K. siamensis*	*K. martiniae*	*K. africana*	*K. jinghongensis*	*K. spencerorum*	*K. piceae*	*K. lodderae*	*K. gamospora*	*K. hellenica*	K. psychrophila	*K. kunashirensis*	*K. exigua*	*K. bulderi*	*K. turicensis*	*K. saulgeensis*	*K. barnettii*
K. viticola	-																										
K. bromeliacearum	23.9	-																									
K. sinensis	23.0	31.9	-																								
K. naganishii	21.6	29.7	86.3	-																							
K. taianensis	21.5	25.3	25.6	25.1	-																						
K. unispora	22.3	28.3	27.9	27.8	27.9	-																					
K. yasuniensis	17.9	30.0	28.0	28.3	28.9	58.5	-																				
K. servazzi	19.5	29.0	26.5	26.9	32.2	55.0	56.8	-																			
K. solicola	17.7	26.7	25.9	27.7	30.4	52.1	54.9	68.2	-																		
K. aerobia	20.7	27.8	27.0	28.4	30.0	53.7	53.7	66.4	75.9	-																	
K. aquatica	23.4	32.4	28.8	28.6	28.4	51.4	53.2	51.5	49.7	48.6	-																
K. siamensis	23.9	25.9	28.7	27.6	30.3	44.5	45.6	45.0	43.5	42.5	56.3	-															
K. martiniae	23.5	20.2	20.9	20.6	19.9	20.2	24.7	23.0	20.6	21.4	22.5	20.6	-														
K. africana	23.6	23.3	24.2	23.5	18.7	18.5	21.7	20.5	19.0	21.8	22.7	22.9	30.2	-													
K. jinghongensis	22.7	19.4	16.9	18.2	17.2	17.1	17.8	18.0	17.8	18.9	18.2	15.6	22.7	22.7	-												
K. spencerorum	20.6	22.9	18.1	19.0	16.5	16.9	16.4	17.7	18.7	19.8	19.9	17.3	25.0	22.8	49.8	-											
K. piceae	20.2	22.0	23.8	25.2	19.5	19.8	19.3	23.6	22.1	21.6	24.2	23.7	26.4	24.8	22.6	23.6	-										
K. lodderae	26.7	20.6	22.7	22.2	19.9	18.8	15.9	18.9	18.4	18.8	19.4	19.2	26.8	26.6	29.9	27.4	26.8	-									
K. gamospora	23.4	18.3	18.1	17.1	15.6	17.6	15.3	17.2	15.2	17.7	18.9	19.7	23.3	25.5	26.8	25.1	25.1	31.6	-								
K. hellenica	22.6	19.5	22.1	21.6	16.7	19.3	17.3	18.6	16.2	18.8	20.9	19.0	22.7	25.6	25.5	23.6	26.4	30.1	52.9	-							
K. psychrophila	22.2	22.2	25.1	25.6	21.1	21.7	20.5	19.3	21.5	22.3	22.5	23.2	25.8	21.5	25.1	27.3	25.9	26.1	29.8	27.4	-						
K. kunashirensis	21.0	20.8	21.9	20.3	20.0	21.9	20.4	18.4	19.7	19.3	22.3	22.2	23.8	23.0	26.9	25.3	24.9	27.8	26.6	29.7	40.3	-					
K. exigua	19.3	20.8	22.8	24.7	19.5	19.4	19.2	17.3	19.5	20.2	20.5	21.7	21.7	21.8	24.6	24.4	25.5	26.6	26.0	27.4	37.6	40.4	-				
K. bulderi	19.7	22.5	23.5	24.9	21.0	20.5	20.0	20.8	21.4	21.7	22.3	23.6	23.2	22.7	24.8	22.7	26.1	26.1	26.2	27.5	36.9	40.4	79.7	-			
K. turicensis	19.1	22.0	24.3	25.3	20.0	20.1	19.7	20.5	21.0	21.7	21.6	22.9	22.9	24.0	24.6	24.1	25.6	26.8	25.1	27.4	35.2	40.1	78.5	91.2	-		
K. saulgeensis	19.5	20.3	23.0	21.4	22.5	19.4	20.8	19.8	19.1	21.2	20.5	23.1	23.3	22.0	25.2	23.7	26.1	28.4	28.0	27.8	37.9	42.1	56.3	56.3	54.7	-	
K. barnettii	20.1	21.2	23.8	22.8	22.0	20.0	21.5	21.6	21.9	22.5	21.7	25.5	22.5	23.6	25.5	22.7	23.5	29.1	28.1	28.8	35.5	40.7	57.2	55.9	54.0	71.7	-

Group 4	*N. dairenensis*	*N. castellii*	*N. baii*
N. dairenensis	-		
N. castellii	36.5	-	
N. baii	33.7	46.9	-

Group 5	*T. iriomotensis*	*T. namnaonensis*	*T. blattae*	*V. polyspora*	*V. tropicalis*	*V. yarrowii*	*V. verrucispora*
T. iriomotensis	-						
T. namnaonensis	34.8	-					
T. blattae	19.2	18.6	-				
V. polyspora	23.2	20.9	18.6	-			
V. tropicalis	22.9	18.7	18.9	25.5	-		
V. yarrowii	20.2	22.4	22.1	26.0	30.6	-	
V. verrucispora	24.2	18.3	19.2	23.8	26.5	35.9	-

Figure. S1
