# Supplementary material for: Functional diversification across yeast lineages of the nucleus-vacuole junction forming protein Nvj1
Source: bioRxiv. 2026 Sep 10:2026.09.05.749613. Preprint. [Version 1] doi: 10.64898/2026.09.05.749613 (PMC13622503; doi:10.64898/2026.09.05.749613)
Supplement: Supplementary file 5 [file media-4.pdf]

Kramer et al.

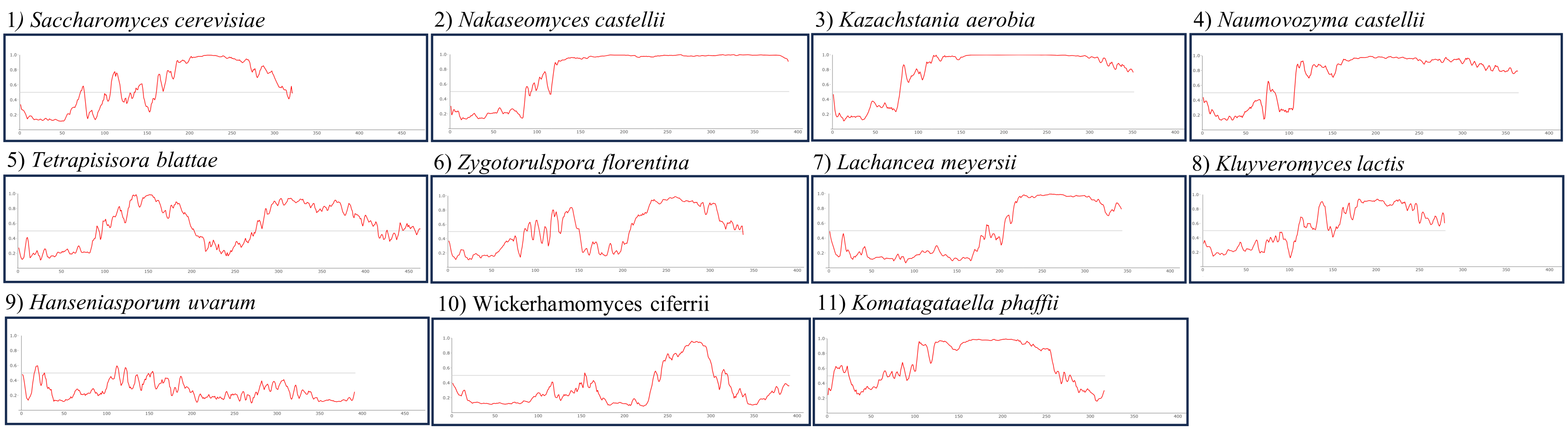

Figure. S4
