# Supplementary material for: Functional diversification across yeast lineages of the nucleus-vacuole junction forming protein Nvj1
Source: bioRxiv. 2026 Sep 10:2026.09.05.749613. Preprint. [Version 1] doi: 10.64898/2026.09.05.749613 (PMC13622503; doi:10.64898/2026.09.05.749613)
Supplement: Supplementary file 10 [file media-9.pdf]

Species Name	seqID	HMMProfileID	Y1000_TreeID	Y1000_DatasetID	Y1000_protID	ncbiID	group	inTableS1	inY1000Hmmer	CopyNumber	syntenyCheck	syntenyComments
saccharomycescerevisiae	saccharomyces_cerevisiae	YHR195W	Saccharomyces_cerevisiae	saccharomyces_cerevisiae	g005344.m1	NP_012065.3	Saccharomyces	1	1	1	MDM31-NVJ1-UTP9	
saccharomycesjurei	saccharomyces_jurei	#N/A	Saccharomyces_jurei	saccharomyces_jurei	g004292.m1	#N/A	Saccharomyces	1	1	1	MDM31-NVJ1-UTP9	
saccharomycesparadoxus	saccharomyces_paradoxus	genemark-Spar_8-processed-gene-4.108	Saccharomyces_paradoxus	saccharomyces_paradoxus	g005237.m1	#N/A	Saccharomyces	1	1	1	MDM31-NVJ1-UTP9	
saccharomyceskudriavzevii	saccharomyces_kudriavzevii	augustus_masked-Skud_8-processed-gene-3.158	Saccharomyces_kudriavzevii	saccharomyces_kudriavzevii	g005184.m1	EJT43435.1	Saccharomyces	1	1	1	MDM31-NVJ1-UTP9	
saccharomycesarboricola	saccharomyces_arboricola	augustus_masked-NC_026178.1-processed-gene-4.111	Saccharomyces_arboricola	saccharomyces_arboricola	g005224.m1	EJS43367.1	Saccharomyces	1	1	1	MDM31-NVJ1-UTP9	
saccharomycesmikatae	saccharomyces_mikatae	snap_masked-Smik_8-processed-gene-4.133	Saccharomyces_mikatae	saccharomyces_mikatae	g005189.m1	#N/A	Saccharomyces	1	1	1	MDM31-NVJ1-UTP9	
saccharomycesuvarum	saccharomyces_uvarum	augustus_masked-Sbay_15-processed-gene-6.183	Saccharomyces_uvarum	saccharomyces_uvarum	g002217.m1	#N/A	Saccharomyces	1	1	1	MDM31-NVJ1-UTP9	
saccharomyceseubayanus	saccharomyces_eubayanus	augustus_masked-chrXV-processed-gene-6.90	Saccharomyces_eubayanus	saccharomyces_eubayanus	g005249.m1	XP_018221971.1	Saccharomyces	1	1	1	MDM31-NVJ1-UTP9	
Nakasesomycesglabrata	Nakasesomyces_glabrata	#N/A	Nakaseomyces_glabratus	yHMPu5000034723_candida_glabrata_180604	g000308.m1	XP_449661.1	Nakasesomyces	1	1	1	MDM31-NVJ1-UTP9	
Nakaseomyceskungkrabaensis	Nakaseomyces_kungkrabaensis	#N/A	Nakaseomyces_kungkrabaensis	yHMPu5000034722_candida_kungkrabaensis_180604	g004198.m1	#N/A	Nakasesomyces	1	1	1	MDM31-NVJ1-UTP9	
Nakaseomycesnivariensis	Nakaseomyces_nivariensis	snap_masked-CANI0S17-processed-gene-4.132	Nakaseomyces_nivariensis	yHMPu5000034720_candida_nivariensis_180604	g001471.m1	#N/A	Nakasesomyces	1	1	1	MDM31-NVJ1-UTP9	
Nakaseomycesuthaithanina	Nakaseomyces_uthaithanina	#N/A	Nakaseomyces_uthaithaninus	yHMPu5000034718_candida_uthaithanina_180604	g005380.m1	#N/A	Nakasesomyces	1	1	1	UTP9?-NVJ1-RTT105?	
nakaseomycesdelphensis	nakaseomyces_delphensis	augustus_masked-NADE0S27-processed-gene-3.39	Nakaseomyces_delphensis	yHMPu5000026127_nakaseomyces_delphensis_160519	g004209.m1	CAO98811.1	Nakasesomyces	1	1	1	MDM31-NVJ1-UTP9	
nakaseomycesbracarensis	nakaseomyces_bracarensis	snap_masked-CABR0S29-processed-gene-6.0	Nakaseomyces_bracarensis	yHMPu5000034725_candida_bracarensis_160928	g001614.m1	#N/A	Nakasesomyces	1	1	1	MDM31-NVJ1-UTP9	
nakaseomycesbacillisporus	nakaseomyces_bacillisporus	augustus_masked-NABA0S29-processed-gene-0.108	Nakaseomyces_bacillisporus	yHMPu5000035666_nakaseomyces_bacillisporus_160613	g004314.m1	#N/A	Nakasesomyces	1	1	1	MDM31-NVJ1-UTP9	
nakaseomycescastellii_	nakaseomyces_castellii_	snap_masked-CACA0S33-processed-gene-9.107	Nakaseomyces_castellii	yHMPu5000034724_candida_castellii_180604	g001576.m1	#N/A	Nakasesomyces	1	1	1	MDM31-NVJ1-UTP9	YGOB disagrees
Nakaseomyces_sp._yHDO568	Nakaseomyces_sp._yHDO568	N/A	Nakaseomyces_sp._yHDO568	yHDO568_nakaseomyces_sp_190924	g004296.m1	N/A	Nakasesomyces	0	1	1	MDM31-NVJ1-UTP9	
kazachstaniasolicola	kazachstania_solicola	augustus_masked-scf7180000046332-processed-gene-1.206	Kazachstania_solicola	yHAB159_kazachstania_solicola_160519.haplomerger2	g005068.m1	#N/A	Kazachstania	1	0	1	MDM31-NVJ1-RIX1?	UTP9 pseudpgenized?
kazachstaniaaerobia	kazachstania_aerobia	augustus_masked-scf7180000020169-processed-gene-0.188	Kazachstania_aerobia	yHAB164_kazachstania_aerobia_160519	g002718.m1	#N/A	Kazachstania	1	0	1	MDM31-NVJ1-RIX1?	needs validation
kazachstaniaunispora	kazachstania_unispora	snap_masked-NODE_47_length_78712_cov_50.1529_ID_93-processed-gene-0.49	Kazachstania_unispora	yHAB133_kazachstania_unispora_160519	g003649.m1	#N/A	Kazachstania	1	0	1	MDM31-NVJ1-RIX1?	needs validation
kazachstania_bromeliacearum	kazachstania_bromeliacearum	snap_masked-NODE_10_length_278180_cov_22.5073_ID_19-processed-gene-1.127	Kazachstania_bromeliacearum	yHAB136_kazachstania_bromeliacearum_160519	g000451.m1	#N/A	Kazachstania	1	1	1	MDM31-NVJ1-RIX1?	needs validation
kazachstania_bulderi	kazachstania_bulderi	#N/A	Kazachstania_bulderi	yHAB157_kazachstania_bulderi_160519.haplomerger2	g005958.m1	#N/A	Kazachstania	1	1	1	MDM31-NVJ1-RIX1?	needs validation
kazachstania_exigua	kazachstania_exigua	#N/A	Kazachstania_exigua	yHAB150_kazachstania_exigua_160519	g002519.m1	#N/A	Kazachstania	1	1	2	MDM31-NVJ1-RIX1?	needs validation
kazachstania_exigua	kazachstania_exigua	#N/A	Kazachstania_exigua	yHAB150_kazachstania_exigua_160519	g000048.m1	#N/A	Kazachstania	0	1	2	MDM31-NVJ1-MRX19	needs validation
kazachstania_gamospora	kazachstania_gamospora	#N/A	Kazachstania_gamospora	yHAB142_kazachstania_gamospora_170307.haplomerger2	g004167.m1	#N/A	Kazachstania	1	1		MCM31-NVJ1-?	contig breakpoint in y1000 main assembly
kazachstania_hellenica	kazachstania_hellenica	#N/A	Kazachstania_hellenica	yHAB145_kazachstania_hellenica_160519.haplomerger2	g003117.m1	#N/A	Kazachstania	1	1		MDM31-NVJ1-RIX1?	
kazachstania_aquatica	Missing!	#N/A	Kazachstania_aquatica	yHAB165_kazachstania_aquatica_160519	g003032.m1	#N/A	Kazachstania	1	1		MDM31-NVJ1-RIX1?	
kazachstania_kunashirensis	kazachstania_kunashirensis	snap_masked-scf7180000008985-processed-gene-1.61	Kazachstania_kunashirensis	yHAB160_kazachstania_kunashirensis_160519	g005050.m1	#N/A	Kazachstania	1	1		MDM31-NVJ1-RIX1?	
kazachstania_lodderae	kazachstania_lodderae	#N/A	Kazachstania_lodderae	yHAB138_kazachstania_lodderae_160519	g003605.m1	#N/A	Kazachstania	1	1		MDM31-NVJ1-RIX1?	
kazachstania_piceae	kazachstania_piceae	#N/A	Kazachstania_piceae	yHAB156_kazachstania_piceae_160519	g005225.m1	#N/A	Kazachstania	1	1		MDM31-NVJ1-RIX1?	
kazachstania_saulgeensis	kazachstania_saulgeensis	#N/A	Kazachstania_saulgeensis	yHMPu5000037225_kazachstania_saulgeensis_210210	g000550.m1	SMN19139.1	Kazachstania	1	1		MDM31-NVJ1-RIX1?	
kazachstania_servazzi	kazachstania_servazzi	#N/A	Kazachstania_servazzii	yHAB151_kazachstania_servazzi_160519	g000773.m1	#N/A	Kazachstania	1	1		MDM31-NVJ1-RIX1?	
kazachstania_sinensis	kazachstania_sinensis	#N/A	Kazachstania_sinensis	yHAB161_kazachstania_sinensis_160519	g005599.m1	#N/A	Kazachstania	1	1		MDM31-NVJ1-RIX1?	
kazachstania_yasuniensis	kazachstania_yasuniensis	#N/A	Kazachstania_yasuniensis	yHMPu5000034708_kazachstania_yasuniensis_180604	g001209.m1	#N/A	Kazachstania	1	1		MDM31-NVJ1-RIX1?	
Kazachstania_barnettii	Kazachstania_barnettii	#N/A	Kazachstania_barnettii	yHAB162_Kazachstania_barnettii_SPADES	g001263.m1	#N/A	Kazachstania	1	1		MDM31-NVJ1-RIX1?	
Kazachstania_psychrophila	Kazachstania_psychrophila	#N/A	Kazachstania_psychrophila	yHMPu5000034706_Kazachstania_psychrophila_SPADES	g002942.m1	#N/A	Kazachstania	1	1		MDM31-NVJ1-RIX1?	
Kazachstania_jinghongensis	Kazachstania_jinghongensis	#N/A	Kazachstania_jinghongensis	yHMPu5000037210_Kazachstania_jinghongensis_SPADES	g001801.m1	#N/A	Kazachstania	1	1		MDM31-NVJ1-RIX1?	
kazachstaniasiamensis	kazachstania_siamensis	augustus_masked-NODE_61_length_63804_cov_14.6622_ID_121-processed-gene-0.80	Kazachstania_siamensis	yHAB143_kazachstania_siamensis_160519	g004344.m1	#N/A	Kazachstania	1	1		MDM31-NVJ1-RIX1?	
kazachstaniataianensis	kazachstania_taianensis	augustus_masked-NODE_5_length_452406_cov_49.1731_ID_9-processed-gene-3.120	Kazachstania_taianensis	yHAB147_kazachstania_taianensis_160519	g004007.m1	#N/A	Kazachstania	1	1		MDM31-NVJ1-RIX1?	
kazachstanianaganishii	kazachstania_naganishii	snap_masked-HE978326-processed-gene-0.118	Kazachstania_naganishii	kazachstania_naganishii	g001440.m1	XP_022467125.1	Kazachstania	1	1		MDM31-NVJ1-RIX1?	
kazachstaniamartiniae	kazachstania_martiniae	snap_masked-scf7180000030514-processed-gene-0.103	Kazachstania_martiniae	yHAB132_kazachstania_martiniae_160519	g004439.m1	#N/A	Kazachstania	1	1		MDM31-NVJ1-RIX1?	
kazachstaniaturicensis	kazachstania_turicensis	snap_masked-scf7180000049581-processed-gene-4.63	Kazachstania_turicensis	yHMPu5000040961_kazachstania_turicensis_201018	g004713.m1	#N/A	Kazachstania	1	1		MDM31-NVJ1-RIX1?	
kazachstaniakunashirensis	kazachstania_kunashirensis	snap_masked-scf7180000008985-processed-gene-1.61	Kazachstania_kunashirensis	yHAB160_kazachstania_kunashirensis_160519	g005050.m1	#N/A	Kazachstania	1	1		MDM31-NVJ1-RIX1?	
kazachstaniaspencerorum	kazachstania_spencerorum	snap_masked-scf7180000055600-processed-gene-0.48	Kazachstania_spencerorum	yHAB155_kazachstania_spencerorum_160519	g002874.m1	#N/A	Kazachstania	1	0		MDM31-NVJ1-RIX1?	
kazachstaniaafricana	kazachstania_africana	augustus_masked-scaffold_2-processed-gene-13.205	Kazachstania_africana	yHAB137_kazachstania_africana_160519	g003755.m1	XP_003956120.1	Kazachstania	1	1		MDM31-NVJ1-?	suspicious gap downstream
kazachstaniaviticola	kazachstania_viticola	snap_masked-scf7180000050361-processed-gene-0.67	Kazachstania_viticola	yHAB158_kazachstania_viticola_160519.haplomerger2	g001183.m1	#N/A	Kazachstania	1	1		MDM31-NVJ1-RIX1?	
Kazachtania_humilis	Kazachstania_humilis		Kazachstania_humilis	yHAB152_candida_humilis_160519.haplomerger2	g000693.m1			0	1		MDM31-NVJ1-RIX1?	
Kazachtania_pseudohumilis	Kazachtania_pseudohumilis		Kazachtania_pseudohumilis	yHMPu5000034719_candida_pseudohumilis_180604.haplomerger2	g002365.m1			0	1		MDM31-NVJ1-RIX1?	
Kazachstania_rosinii	Kazachstania_rosinii		Kazachstania_rosinii	yHAB153_kazachstania_rosinii_160519	g001213.m1			0	1		MDM31-NVJ1-?	downstream gene is small
Kazachstania_sp._UFMG-CM-Y273	Kazachstania_sp._UFMG-CM-Y273		Kazachstania_sp._UFMG-CM-Y273	yHDO576_kazachstania_sp_180604	g000466.m1			0	1		MDM31-NVJ1-RIX1?	
naumovozymadairenensis	naumovozyma_dairenensis	augustus_masked-NC_016482.1-processed-gene-7.22	Naumovozyma_dairenensis	yHMPu5000034872_naumovozyma_dairenensis_180604	g002752.m1	XP_003669889.1	Naumovozyma	1	1		MDM31-NVJ1-RIX1?	
naumovozymacastellii	naumovozyma_castellii	snap_masked-HE576752-processed-gene-12.71	Naumovozyma_castellii	yHMPu5000034871_naumovozyma_castellii_180604	g001098.m1	#N/A	Naumovozyma	1	1		MDM31-NVJ1-RIX1?	
naumovozyma_baii	naumovozyma_baii	#N/A	Naumovozyma_baii	yHMPu5000034894_naumovozyma_baii_190924	g000794.m1	#N/A	Naumovozyma	1	1		MDM31-NVJ1-RIX1?	
tetrapisisporairiomotensis	tetrapisispora_iriomotensis	augustus_masked-flattened_line_57-processed-gene-0.130	Tetrapisispora_iriomotensis	yHMPu5000034876_Tetrapisispora_iriomotensis_SPADES	g003416.m1	#N/A	Tetrapisispora	1	1		MDM31-NVJ1-NMD5-UTP9	
tetrapisisporanamnaonensis	tetrapisispora_namnaonensis	#N/A	Tetrapisispora_namnaoensis	yHMPu5000034877_tetrapisispora_namnaonensis_160519	g001993.m1	#N/A	Tetrapisispora	1	0		MDM31-NVJ1-NMD5-UTP9	synteny graph hits wrong MDM31
tetrapisisporablattae	tetrapisispora_blattae	snap_masked-NC_020185-processed-gene-25.62	Tetrapisispora_blattae	yHMPu5000034874_tetrapisispora_blattae_190924	g003018.m1	XP_004178357.1	Tetrapisispora	1	0		PEX14-NVJ1-UTP9	No MDM31 in vicinity
vanderwaltozymapolyspora	vanderwaltozyma_polyspora	snap_masked-NW_001834637.1-processed-gene-0.82	Vanderwaltozyma_polyspora	yHMPu5000034869_vanderwaltozyma_polyspora_180604	g005365.m1	XP_001643905.1	Vanderwaltozyma	1	1		MDM31-NVJ1-NMD5-UTP9	
Vanderwaltozyma_yarrowii	Vanderwaltozyma_yarrowii	#N/A	Vanderwaltozyma_yarrowii	yHMPu5000034868_Vanderwaltozyma_yarrowii_S33_SPADES	g001449.m1	#N/A	Vanderwaltozyma	1	1		MDM31-NVJ1-NMD5-UTP9	
vanderwaltozyma_tropicalis	vanderwaltozyma_tropicalis	#N/A	Vanderwaltozyma_tropicalis	yHMPu5000026257_vanderwaltozyma_tropicalis_190924	g003717.m1	#N/A	Vanderwaltozyma	1	1		MDM31-NVJ1-NMD5-UTP9	
Vanderwaltozyma_verrucispora	Vanderwaltozyma_verrucispora	#N/A	Vanderwaltozyma_verrucispora	yHMPu5000037837_Vanderwaltozyma_verrucispora_S176_SPADES	g005143.m1	#N/A	Vanderwaltozyma	1	1		MDM31-NVJ1-NMD5-UTP9	
torulasporapretoriensis	torulaspora_pretoriensis	augustus_masked-flattened_line_3-processed-gene-4.12	Torulaspora_pretoriensis	yHMPu5000034881_Torulaspora_pretoriensis_SPADES	g004938.m1	#N/A	Torulaspora	1	1		MDM31-NVJ1-NMD5-UTP9	
torulasporamicroellipsoides	torulaspora_microellipsoides	augustus_masked-flattened_line_52-processed-gene-0.132	Torulaspora_microellipsoides	yHMPu5000035651_Torulaspora_microellipsoides_SPADES	g002639.m1	#N/A	Torulaspora	1	1		MDM31-NVJ1-FEN2?	weird gap after MDM31
torulasporafranciscae	torulaspora_franciscae	snap_masked-flattened_line_10-processed-gene-1.40	Torulaspora_franciscae	yHMPu5000026152_Torulaspora_franciscae_SPADES	g004752.m1	#N/A	Torulaspora	1	1		MDM31-NVJ1-NMD5-UTP9	
torulasporadelbrueckii	torulaspora_delbrueckii	snap_masked-scaffold_4-processed-gene-1.34	Torulaspora_delbrueckii	yHMPu5000035653_torulaspora_delbrueckii_160613	g001776.m1	XP_003680850.1	Torulaspora	1	1		MDM31-NVJ1-NMD5-UTP9	
torulaspora_globosa	torulaspora_globosa	#N/A	Torulaspora_globosa	yHMPu5000034880_torulaspora_globosa_160519	g001826.m1	#N/A	Torulaspora	1	1		MDM31-NVJ1-NMD5-UTP9	
torulaspora_maleeae	torulaspora_maleeae	snap_masked-NODE_5_length_764704_cov_59.617_ID_1253-processed-gene-6.109	Torulaspora_maleeae	yHMPu5000035652_torulaspora_maleeae_160613	g003867.m1	#N/A	Torulaspora	1	1		MDM31-NVJ1-NMD5-UTP9	
Torulaspora_sp._yHMJ407	Torulaspora_sp._yHMJ407		Torulaspora_sp._yHMJ407	yHMJ407_Torulaspora_sp_nov_plate34_SPADES	g002906.m1			0	1		MDM31-NVJ1-NMD5-UTP9	
zygosaccharomyces_bisporus	zygosaccharomyces_bisporus	snap_masked-flattened_line_16-processed-gene-0.27	Zygosaccharomyces_bisporus	yHMPu5000034866_zygosaccharomyces_bisporus_160519	g005101.m1	#N/A	Zygosaccharomyces	1	1		MDM31-NVJ1-NMD5-UTP9	
zygosaccharomyces_gambellarensis	zygosaccharomyces_gambellarensis	#N/A	Zygosaccharomyces_gambellarensis	yHDO565_zygosaccharomyces_gambellarensis_180604	g005022.m1	#N/A	Zygosaccharomyces	1	1		MDM31-NVJ1-NMD5-UTP9	
zygosaccharomycesbailii	Zygosaccharomyces_bailii	augustus_masked-ZYBA0S10-processed-gene-3.53	Zygosaccharomyces_bailii	zygosaccharomyces_bailii	g001048.m1	CDF91308.1	Zygosaccharomyces	1	1		MDM31-NVJ1-NMD5-UTP9	

Species Name	seqID	HMMProfileID	Y1000_TreeID	Y1000_DatasetID	Y1000_protID	ncbiID	group	inTableS1	inY1000Hmmer	Copy Number	syntenyCheck	syntenyComments
zygosaccharomyceskombuchaensis	Zygosaccharomyces_kombuchaensis	snap_masked-NODE_1_length_271579_cov_10.1807_ID_1-processed-gene-1.23	Zygosaccharomyces_kombuchaensis	yHMPu5000034865_zygosaccharomyces_kombuchaensis_160519	g000063.m1	#N/A	Zygosaccharomyces	1	1		MDM31-NVJ1-NMD5-UTP9	
zygosaccharomyces_lentus	zygosaccharomyces_lentus	#N/A	Zygosaccharomyces_lentus	yHMPu5000034864_zygosaccharomyces_lentus_170307	g004388.m1	#N/A	Zygosaccharomyces	1	1		MDM31-NVJ1-NMD5-UTP9	
zygosaccharomyces_mellis	zygosaccharomyces_mellis	#N/A	Zygosaccharomyces_mellis	yHDO572_zygosaccharomyces_mellis_180604	g002135.m1	#N/A	Zygosaccharomyces	1	1		MDM31-NVJ1-NMD5-UTP9	
zygosaccharomyces_pseudorouxii	zygosaccharomyces_pseudorouxii	#N/A	Zygosaccharomyces_pseudorouxii	yHMPu5000037836_zygosaccharomyces_pseudorouxii_201018.haplomerger2	g004410.m1	#N/A	Zygosaccharomyces	1	1		MDM31-NVJ1-NMD5-UTP9	
zygosaccharomyces_siamensis	zygosaccharomyces_siamensis	#N/A	Zygosaccharomyces_siamensis	yHMPu5000035626_zygosaccharomyces_siamensis_190924	g000372.m1	#N/A	Zygosaccharomyces	1	1		MDM31-NVJ1-NMD5-UTP9	
Zygosaccharomyces_parabailii	Zygosaccharomyces_parabailii		Zygosaccharomyces_parabailii	yHMPu5000037834_zygosaccharomyces_parabailii_190924	g003412.m1			0	1		MDM31-NVJ1-NMD5-UTP9	
Zygosaccharomyces_pseudobailii	Zygosaccharomyces_pseudobailii		Zygosaccharomyces_pseudobailii	yHMPu5000035627_zygosaccharomyces_pseudobailii_201018	g002625.m1			0	1		MDM31-NVJ1-?	small contig
Zygosaccharomyces_pseudobailii	Zygosaccharomyces_pseudobailii		Zygosaccharomyces_pseudobailii	yHMPu5000035627_zygosaccharomyces_pseudobailii_201018	g003112.m1			0	1		?-NVJ1-NMD5	
Zygosaccharomyces_rouxii	Zygosaccharomyces_rouxii		Zygosaccharomyces_rouxii	yHMPu5000034863_zygosaccharomyces_rouxii_180604	g001572.m1			0	1		MDM31-NVJ1-NMD5-UTP9	
Zygosaccharomyces_sapae	Zygosaccharomyces_sapae		Zygosaccharomyces_sapae	yHDO603_zygosaccharomyces_sapae_190924.haplomerger2	g000017.m1			0	1		MDM31-NVJ1-NMD5-UTP9	
zygotorulasporaflorentina	zygotorulaspora_florentina	augustus_masked-NODE_6_length_562868_cov_24.3835_ID_11-processed-gene-2.47	Zygotorulaspora_florentina	yHMPu5000034862_zygotorulaspora_florentina_160519	g004327.m1	#N/A	Zygotorulaspora	1	1		MDM31-NVJ1-NMD5-UTP9	
zygotorulasporamrakii	zygotorulaspora_mrakii	snap_masked-NODE_2_length_708340_cov_18.0358_ID_2767-processed-gene-4.105	Zygotorulaspora_mrakii	yHMPu5000026256_zygotorulaspora_mrakii_170307	g002003.m1	#N/A	Zygotorulaspora	1	1		MDM31-NVJ1-NMD5-UTP9	
zygotorulaspora_chibaensis	zygotorulaspora_chibaensis	#N/A	Zygotorulaspora_chibaensis	yHMPu5000037204_zygotorulaspora_chibaensis_210210	g002973.m1	#N/A	Zygotorulaspora	1	1		MDM31-NVJ1-NMD5-UTP9	
zygotorulaspora_danielsina	zygotorulaspora_danielsina	#N/A	Zygotorulaspora_danielsina	yHMPu5000037205_zygotorulaspora_danielsina_210210	g002121.m1	#N/A	Zygotorulaspora	1	1		MDM31-NVJ1-NMD5-UTP9	
zygotorulaspora_mrakii	zygotorulaspora_mrakii	snap_masked-NODE_2_length_708340_cov_18.0358_ID_2767-processed-gene-4.105	Zygotorulaspora_mrakii	yHMPu5000026256_zygotorulaspora_mrakii_170307	g002003.m1	#N/A	Zygotorulaspora	1	1		MDM31-NVJ1-NMD5-UTP9	
Zygotorulaspora_sp._yHDO592	Zygotorulaspora_sp._yHDO592		Zygotorulaspora_sp._yHDO592	yHDO592_zygotorulaspora_sp_190924	g004529.m1			0	1		MDM31-NVJ1-NMD5-UTP9	
hagleromyces_aurorensis	hagleromyces_aurorensis	#N/A	Hagleromyces_aurorensis	yHDO579_hagleromyces_aurorensis_180604	g002965.m1	#N/A	Zygotorulaspora	1	1		MDM31-NVJ1-NMD5-UTP9	
lachanceakluyveri	lachancea_kluyveri	snap_masked-SAKL0G-processed-gene-15.78	Lachancea_kluyveri	yHMPu5000034694_lachancea_kluyveri_180604	g001117.m1	#N/A	Lachancea	1	1		MDM31-NVJ1-NMD5-UTP9	
lachancea_mirantina	lachancea_mirantina	augustus_masked-LAMI0C-processed-gene-0.22	Lachancea_mirantina	yHMPu5000034691_lachancea_mirantina_180604	g001548.m1	SCU82809.1	Lachancea	1	1		MDM31-NVJ1-NMD5-?	
Lachancea_nothofagi	Lachancea_nothofagi	snap_masked-LANO0B-processed-gene-1.74	Lachancea_nothofagi	yHMPu5000034690_lachancea_nothofagi_180604	g001406.m1	SCU80770.1	Lachancea	1	1		MDM31-NVJ1-NMD5-UTP9	
lachanceafantastica	lachancea_fantastica	#N/A	Lachancea_fantastica_nom._nud.	lachancea_fantastica	g003435.m1	SCU92434.1	Lachancea	1	1		MDM31-NVJ1-NMD5-UTP9	
lachancealanzarotensis	lachancea_lanzarotensis	augustus_masked-LN736361.1-processed-gene-3.12	Lachancea_lanzarotensis	yHMPu5000034693_Lachancea_lanzarotensis_SPADES	g004947.m1	XP_022627210.1	Lachancea	1	1		MDM31-NVJ1-NMD5-UTP9	
lachanceawaltii	lachancea_waltii	snap_masked-AADM01000119.1-processed-gene-0.11	Lachancea_waltii	yHMPu5000026219_lachancea_waltii_180604	g003199.m1	#N/A	Lachancea	1	1		MDM31-NVJ1-NMD5-UTP9	
lachanceaquebecensis	lachancea_quebecensis	snap_masked-LAQU0S22-processed-gene-0.116	Lachancea_quebecensis	yHMPu5000040963_lachancea_quebecuensis_200128	g002616.m1	#N/A	Lachancea	1	1		MDM31-NVJ1-NMD5-UTP9	
lachanceathermotolerans	lachancea_thermotolerans	snap_masked-CU928168-processed-gene-14.65	Lachancea_thermotolerans	yHMPu5000034678_lachancea_thermotolerans_180604	g003213.m1	XP_002553478.1	Lachancea	1	1		MDM31-NVJ1-NMD5-UTP9	
lachanceameyersii	lachancea_meyersii	snap_masked-LAME0G-processed-gene-17.24	Lachancea_meyersii	yHMPu5000034692_lachancea_meyersii_180604	g001214.m1	SCV01664.1	Lachancea	1	1		MDM31-NVJ1-NMD5-UTP9	
lachanceadasiensis	lachancea_dasiensis	augustus_masked-LADA0A-processed-gene-7.6	Lachancea_dasiensis	yHMPu5000034697_lachancea_dasiensis_190924	g003259.m1	SCU78828.1	Lachancea	1	1		MDM31-NVJ1-NMD5-UTP9	
lachanceafermentati	lachancea_fermentati	augustus_masked-LAFE0A-processed-gene-1.131	Lachancea_fermentati	yHMPu5000034695_lachancea_fermentati_201018	g004518.m1	SCV99344.1	Lachancea	1	1		MDM31-NVJ1-NMD5-UTP9	
Lachancea_cidri_NRRL_Y-12635	Lachancea_cidri_NRRL_Y-12635		Lachancea_cidri_NRRL_Y-12635	lookup				0	1		MDM31-NVJ1-NMD5-UTP9	
eremotheciugossypii	eremotheciu_gossypii	#N/A	Eremothecium_gossypii	eremothecium_gossypii	NA	NP_982880.2	Eremothecium	1	0		MDM31-NMD5-UTP9	NVJ1 not annotated in y1000
eremotheciumcymbalariae	eremothecium_cymbalariae	snap_masked-NC_016453.1-processed-gene-3.121	Eremothecium_cymbalariae	eremothecium_cymbalariae	g002832.m1	XP_003646754.1	Eremothecium	1	1		MDM31-NVJ1-NMD5-UTP9	
eremotheciumcoryli	eremothecium_coryli	maker-AZAH01000009.1-snap-gene-0.44	Eremothecium_coryli	eremothecium_coryli	g004362.m1	#N/A	Eremothecium	1	0		MDM31-NVJ1-NMD5-UTP9	
eremotheciumsinecaudum	eremothecium_sinecaudum	#N/A	Eremothecium_sinecaudum	eremothecium_sinecaudum	g003584.m1	XP_017989102.1	Eremothecium	1	1		MDM31-NVJ1-NMD5-?	
kluyveromyceslactis	kluyveromyces_lactis	#N/A	Kluyveromyces_lactis_CBS_2359	kluyveromyces_lactis	g000677.m1	XP_451721.1	Kluyveromyces	1	1		MDM31-NVJ1-NMD5-UTP9	
Kluyveromyces_sp._yHMH660	Kluyveromyces_sp._yHMH660		Kluyveromyces_sp._yHMH660	yHMH660_Kluyveromyces_sp_nov_plate33_SPADES	g005129.m1			0	1		MDM31-NVJ1-NMD5-UTP9	
kluyveromycesdobzhanskii	kluyveromyces_dobzhanskii	snap_masked-NODE_13_length_251415_cov_20.6991_ID_25-processed-gene-1.144	Kluyveromyces_dobzhanskii	yHMPu5000034710_kluyveromyces_dobzhanskii_160519	g001256.m1	CDO92201.1	Kluyveromyces	1	1		MDM31-NVJ1-NMD5-UTP9	
Kluyveromyces_aestuarii_NRRL_YB-4510	Kluyveromyces_aestuarii_NRRL_YB-4510		Kluyveromyces_aestuarii_NRRL_YB-4510	yHMPu5000034709_kluyveromyces_aestuarii_160519	g003160.m1			0	1		MDM31-NVJ1-NMD5-UTP9	
kluyveromyces_nonfermentans	kluyveromyces_nonfermentans	#N/A	Kluyveromyces_nonfermentans	yHMPu5000034701_kluyveromyces_nonfermentans_170307	g001343.m1	#N/A	Kluyveromyces	1	1		MDM31-NVJ1-NMD5-UTP9	
kluyveromyces_siamensis	kluyveromyces_siamensis	#N/A	Kluyveromyces_siamensis	yHMPu5000034700_kluyveromyces_siamensis_180604	g003460.m1	#N/A	Kluyveromyces	1	1		MDM31-NVJ1-NMD5-UTP9	
kluyveromyces_starmeri	kluyveromyces_starmeri	#N/A	Kluyveromyces_starmeri	yHDO570_kluyveromyces_starmeri_180604	g003161.m1	#N/A	Kluyveromyces	1	1		MDM31-NVJ1-NMD5-UTP9	
kluyveromyces_wickerhamii	kluyveromyces_wickerhamii	#N/A	Kluyveromyces_wickerhamii	yHMPu5000034699_kluyveromyces_wickerhamii_170307	g000532.m1	#N/A	Kluyveromyces	1	1		MDM31-NVJ1-NMD5-UTP9	
hanseniasporauvarum	hanseniaspora_uvarum	#N/A	Hanseniaspora_uvarum_DSMZ_2768	hanseniaspora_uvarum	g000873.m1	KKA03541.1	Hanseniaspora	1	0		<-NVJ1-MDM31, UTP9	inversion
hanseniasporapseudoguilliermondi	hanseniaspora_pseudoguilliermondi	#N/A	Hanseniaspora_pseudoguilliermondii	yHMPu5000035695_hanseniaspora_pseudoguilliermondii_160519.haplomerger2	g000765.m1	#N/A	Hanseniaspora	1	0		<-NVJ1-MDM31, UTP9	
hanseniasporaclermontiae	hanseniaspora_clermontiae	#N/A	Hanseniaspora_clermontiae	yHMPu5000034963_hanseniaspora_clermontiae_160519.haplomerger2	g003387.m1	#N/A	Hanseniaspora	1	0		<-NVJ1-MDM31, UTP9	
hanseniasporavalbyensis	hanseniaspora_valbyensis	#N/A	Hanseniaspora_valbyensis	yHMPu5000034955_hanseniaspora_valbyensis_201018.haplomerger2	g000788.m1	OBA26588.1	Hanseniaspora	1	0		<-NVJ1-MDM31, UTP9	
hanseniasporavinae	hanseniaspora_vinae	#N/A	Hanseniaspora_vineae	yHMPu5000026147_hanseniaspora_vineae_190924.haplomerger2	g000163.m1	#N/A	Hanseniaspora	1	0		?	small contig
cyberlindnerasuaveolens	cyberlindnera_suaveolens	#N/A	Cyberlindnera_suaveolens	yHMPu5000035687_cyberlindnera_suaveolens_160613	g005674.m1	#N/A	Cyberlindnera	1	0		MDM31-NVJ1-?-NMD5-UTP9	
cyberlindnerasaturnus	cyberlindnera_saturnus	#N/A	Cyberlindnera_saturnus	yHMPu5000035686_Cyberlindnera_saturnus_SPADES	g001009.m1	#N/A	Cyberlindnera	1	0		MDM31-NVJ1-?-NMD5-UTP9	
cyberlindneramisumaiensis	cyberlindnera_misumaiensis	#N/A	Cyberlindnera_misumaiensis	yHMPu5000034979_cyberlindnera_misumaiensis_160519	g002417.m1	#N/A	Cyberlindnera	1	0		MDM31-NVJ1-?-NMD5-?	
Cyberlindnerajadinii	Cyberlindnera_jadinii	#N/A	Cyberlindnera_jadinii	yHMPu5000035336_cyberlindnera_jadinii_180604.haplomerger2	g002673.m1	XP_020072378.1	Cyberlindnera	1	0		?	contig break
Cyberlindnerafabianii	Cyberlindnera_fabianii	#N/A	Cyberlindnera_fabianii	yHMPu5000035701_cyberlindnera_fabianii_160519	g005778.m1	ONH68477.1	Cyberlindnera	1	0		?	MDM31 nearby
cyberlindneramaclurae	cyberlindnera_maclurae	#N/A	Cyberlindnera_maclurae	yHMPu5000035699_cyberlindnera_maclurae_160613	g003717.m1	#N/A	Cyberlindnera	1	1		MDM31-NVJ1-?-NMD5-UTP9	
wickerhamomycesalni	wickerhamomyce_salni	#N/A	Wickerhamomyces_alni	yHMPu5000035274_wickerhamomyces_alni_170307	g001244.m1	#N/A	Wickerhamomyces	1	0		MDM31-NVJ1-?-NMD5-?	
wickerhamomycescanadensis	wickerhamomyces_canadensis	#N/A	Wickerhamomyces_canadensis	yHMPu5000035639_wickerhamomyces_canadensis_160613	g004559.m1	#N/A	Wickerhamomyces	1	0		MDM31-NVJ1-?-NMD5-UTP9	
wickerhamomycesciferrii	wickerhamomyces_ciferrii	#N/A	Wickerhamomyces_ciferrii	yHMPu5000035269_wickerhamomyces_ciferrii_170307	g005482.m1,g003054.m1	XP_011277532.1	Wickerhamomyces	1	0		?	
Wickerhamomyceschambardii	Wickerhamomyces_chambardii	#N/A	Wickerhamomyces_chambardii	yHMPu5000035270_wickerhamomyces_chambardii_180604.haplomerger2	g001027.m1	#N/A	Wickerhamomyces	1	0		<-UTP9, MDM31,<-MVJ1-NMD5	
Wickerhamomyceshampshirensis	Wickerhamomyces_hampshirensis	#N/A	Wickerhamomyces_hampshirensis	yHMPu5000035268_wickerhamomyces_hampshirensis_160928	g002138.m1	#N/A	Wickerhamomyces	1	0		MDM31-NVJ1-?-NMD5-?	
Wickerhamomycesmucosus	Wickerhamomyces_mucosus	#N/A	Wickerhamomyces_mucosus	yHMPu5000038048_wickerhamomyces_mucosus_170912.haplomerger2	g002404.m1	#N/A	Wickerhamomyces	1	0		?	
Wickerhamomycespiperi	Wickerhamomyces_piperi	#N/A	Wickerhamomyces_pijperi	yHMPu5000035254_wickerhamomyces_pijperi_160928.haplomerger2	g002584.m1	#N/A	Wickerhamomyces	1	0		<-NVJ1, NMD5-MDM31,?-?-?-UTP9	
komagataellaphaffi	komagataella_phaffi	#N/A	Komagataella_phaffii_GS115	komagataella_phaffi	g002262.m1	XP_002491603.1	Komagataella	1	0		MDM31-?,<-NVJ1, NMD5	
komagataellapopuli	komagataella_populi	#N/A	Komagataella_populi	yHMPu5000026274_komagataella_populi_160519	g004657.m1	#N/A	Komagataella	1	0		MDM31-?,<-NVJ1, NMD5	
komagataellapastoris	komagataella_pastoris	#N/A	Komagataella_pastoris_ATCC_28485	komagataella_pastoris	g002679.m1	ANZ75330.1	Komagataella	1	0		MDM31-?,<-NVJ1, NMD5	

Species Name	seqID	HMMProfileID	Y1000_TreeID	Y1000_DatasetID	Y1000_protID	ncbiID	group	inTableS1	inY1000Hmmer	CopyNumber	syntenyCheck	syntenyComments
Komagataellakurtzmanii	*Komagataella_kurtzmanii*	*#N/A*	*Komagataella_kurtzmanii*	*yHMPu5000035676_komagataella_kurtzmanii_160613*	*g004231.m1*	*#N/A*	*Komagataella*	1	0		MDM31-?,<-NVJ1, NMD5	
Komatagataella	Komatagaella_mondaviorum	*#N/A*	Komagataella_mondaviorum	*yHMPu5000037220_komagataella_mondaviorum_210210*	*g004487.m1*	*#N/A*	*Komagataella*	1	0		MDM31-?,<-NVJ1, NMD5	
Kuraishia	Kuraishia_floccosa	*#N/A*	*Kuraishia_floccosa*	*yHMPu5000034935_kuraishia_floccosa_210210*	*g004700.m1*	*#N/A*	Kuraishia	1	0		MDM31-?,<-NVJ1, NMD5	
NA	Asbhya_aceri	*#N/A*	Ashbya_aceri	ashbya_aceri	NA	AGO10388.1	Eremothecium	1	0		MDM31-NMD5-UTP9	No NVJ1 annotation in Y1000 data
Wickerhamomyces_anomalus	Wickerhamomyces_anomalus	*#N/A*	Wickerhamomyces_anomalus	yHMPu5000035273_wickerhamomyces_anomalus_180604.haplomerger2	g001657.m1	TBD	Wickerhamomyces	1	0		MDM31-?,<-NVJ1-?, NMD5	

Species Name	seqID	HMMProfileID	Y1000_TreeID	Y1000_DatasetID	Y1000_protID	ncbiID	group	,	Molecular Weight (KDa)	,	Isoelectric Point			Molecular Weight (Da)
saccharomycescerevisiae	saccharomyces_cerevisiae	YHR195W	Saccharomyces_cerevisiae	saccharomyces_cerevisiae	g005344.m1	NP_012065.3	Saccharomyces	,	36.42145	,	4.7988			36421.45
saccharomycesjurei	saccharomyces_jurei	#N/A	Saccharomyces_jurei	saccharomyces_jurei	g004292.m1	#N/A	Saccharomyces	,	36.85304	,	5.4497			36853.04
saccharomycesparadoxus	saccharomyces_paradoxus	genemark-Spar_8-processed-gene-4.108	Saccharomyces_paradoxus	saccharomyces_paradoxus	g005237.m1	#N/A	Saccharomyces	,	36.47436	,	4.5513			36474.36
saccharomyceskudriavzevii	saccharomyces_kudriavzevii	augustus_masked-Skud_8-processed-gene-3.158	Saccharomyces_kudriavzevii	saccharomyces_kudriavzevii	g005184.m1	EJT43435.1	Saccharomyces	,	36.15633	,	4.7852			36156.33
saccharomycesarboricola	saccharomyces_arboricola	augustus_masked-NC_026178.1-processed-gene-4.111	Saccharomyces_arboricola	saccharomyces_arboricola	g005224.m1	EJS43367.1	Saccharomyces	,	36.45834	,	4.6479			36458.34
saccharomycesmikatae	saccharomyces_mikatae	snap_masked-Smik_8-processed-gene-4.133	Saccharomyces_mikatae	saccharomyces_mikatae	g005189.m1	#N/A	Saccharomyces	,	36.92298	,	4.6452			36922.98
saccharomycesuvarum	saccharomyces_uvarum	augustus_masked-Sbay_15-processed-gene-6.183	Saccharomyces_uvarum	saccharomyces_uvarum	g002217.m1	#N/A	Saccharomyces	,	36.68756	,	4.5178			36687.56
saccharomyceseubayanus	saccharomyces_eubayanus	augustus_masked-chrXV-processed-gene-6.90	Saccharomyces_eubayanus	saccharomyces_eubayanus	g005249.m1	XP_018221971.1	Saccharomyces	,	36.37819	,	4.4885			36378.19
Nakasesomycesglabrata	Nakasesomyces_glabrata	#N/A	Nakaseomyces_glabratus	yHMPu5000034723_candida_glabrata_180604	g000308.m1	XP_449661.1	Nakasesomyces	,	27.2777	,	4.748			27277.7
Nakaseomyceskungkrabaensis	Nakaseomyces_kungkrabaensis	#N/A	Nakaseomyces_kungkrabaensis	yHMPu5000034722_candida_kungkrabaensis_180604	g004198.m1	#N/A	Nakasesomyces	,	26.47484	,	4.4612			26474.84
Nakaseomycesnivariensis	Nakaseomyces_nivariensis	snap_masked-CANI0S17-processed-gene-4.132	Nakaseomyces_nivariensis	yHMPu5000034720_candida_nivariensis_180604	g001471.m1	#N/A	Nakasesomyces	,	26.54481	,	4.4612			26544.81
Nakaseomycesuthaithanina	Nakaseomyces_uthaithanina	#N/A	Nakaseomyces_uthaithaninus	yHMPu5000034718_candida_uthaithanina_180604	g005380.m1	#N/A	Nakasesomyces	,	25.03534	,	5.7115			25035.34
nakaseomycesdelphensis	nakaseomyces_delphensis	augustus_masked-NADE0S27-processed-gene-3.39	Nakaseomyces_delphensis	yHMPu5000026127_nakaseomyces_delphensis_160519	g004209.m1	CAO98811.1	Nakasesomyces	,	26.76638	,	5.0106			26766.38
nakaseomycesbracarensis	nakaseomyces_bracarensis	snap_masked-CABR0S29-processed-gene-6.0	Nakaseomyces_bracarensis	yHMPu5000034725_candida_bracarensis_160928	g001614.m1	#N/A	Nakasesomyces	,	30.75206	,	3.9239			30752.06
nakaseomycesbacillisporus	nakaseomyces_bacillisporus	augustus_masked-NABA0S29-processed-gene-0.108	Nakaseomyces_bacillisporus	yHMPu5000035666_nakaseomyces_bacillisporus_160613	g004314.m1	#N/A	Nakasesomyces	,	30.26465	,	5.5641			30264.65
nakaseomycescastellii_	nakaseomyces_castellii_	snap_masked-CACA0S33-processed-gene-9.107	Nakaseomyces_castellii	yHMPu5000034724_candida_castellii_180604	g001576.m1	#N/A	Nakasesomyces	,	43.0299	,	4.8634			43029.9
kazachstaniasolicola	kazachstania_solicola	augustus_masked-scf7180000046332-processed-gene-1.206	Kazachstania_solicola	yHAB159_kazachstania_solicola_160519.haplomerger2	g005068.m1	#N/A	Kazachstania	,	39.01031	,	3.9829			39010.31
kazachstaniaaerobia	kazachstania_aerobia	augustus_masked-scf7180000020169-processed-gene-0.188	Kazachstania_aerobia	yHAB164_kazachstania_aerobia_160519	g002718.m1	#N/A	Kazachstania	,	40.26682	,	4.1348			40266.82
kazachstaniaunispora	kazachstania_unispora	snap_masked-NODE_47_length_78712_cov_50.1529_ID_93-processed-gene-0.49	Kazachstania_unispora	yHAB133_kazachstania_unispora_160519	g003649.m1	#N/A	Kazachstania	,	38.83565	,	4.1879			38835.65
kazachstania_bromeliacearum	kazachstania_bromeliacearum	snap_masked-NODE_10_length_278180_cov_22.5073_ID_19-processed-gene-1.127	Kazachstania_bromeliacearum	yHAB136_kazachstania_bromeliacearum_160519	g000451.m1	#N/A	Kazachstania	,	26.35025	,	4.4822			26350.25
kazachstania_bulderi	kazachstania_bulderi	#N/A	Kazachstania_bulderi	yHAB157_kazachstania_bulderi_160519.haplomerger2	g005958.m1	#N/A	Kazachstania	,	49.04927	,	4.3063			49049.27
kazachstania_exigua	kazachstania_exigua	#N/A	Kazachstania_exigua	yHAB150_kazachstania_exigua_160519	g002519.m1	#N/A	Kazachstania	,	49.09724	,	4.2164			49097.24
kazachstania_gamospora	kazachstania_gamospora	#N/A	Kazachstania_gamospora	yHAB142_kazachstania_gamospora_170307.haplomerger2	g004167.m1	#N/A	Kazachstania	,	41.89352	,	4.6891			41893.52
kazachstania_hellenica	kazachstania_hellenica	#N/A	Kazachstania_hellenica	yHAB145_kazachstania_hellenica_160519.haplomerger2	g003117.m1	#N/A	Kazachstania	,	47.51685	,	4.232			47516.85
kazachstania_aquatica	Missing!	#N/A	Kazachstania_aquatica	yHAB165_kazachstania_aquatica_160519	g003032.m1	#N/A								
kazachstania_kunashirensis	kazachstania_kunashirensis	snap_masked-scf7180000008985-processed-gene-1.61	Kazachstania_kunashirensis	yHAB160_kazachstania_kunashirensis_160519	g005050.m1	#N/A	Kazachstania	,	34.65823	,	4.3628			34658.23
kazachstania_lodderae	kazachstania_lodderae	#N/A	Kazachstania_lodderae	yHAB138_kazachstania_lodderae_160519	g003605.m1	#N/A	Kazachstania	,	48.90606	,	4.9175			48906.06
kazachstania_piceae	kazachstania_piceae	#N/A	Kazachstania_piceae	yHAB156_kazachstania_piceae_160519	g005225.m1	#N/A	Kazachstania	,	52.16235	,	4.8196			52162.35
kazachstania_saulgeensis	kazachstania_saulgeensis	#N/A	Kazachstania_saulgeensis	yHMPu5000037225_kazachstania_saulgeensis_210210	g000550.m1	SMN19139.1	Kazachstania	,	44.35671	,	4.0589			44356.71
kazachstania_servazzi	kazachstania_servazzi	#N/A	Kazachstania_servazzii	yHAB151_kazachstania_servazzi_160519	g000773.m1	#N/A	Kazachstania	,	36.19702	,	4.0021			36197.02
kazachstania_sinensis	kazachstania_sinensis	#N/A	Kazachstania_sinensis	yHAB161_kazachstania_sinensis_160519	g005599.m1	#N/A	Kazachstania	,	32.84921	,	4.2566			32849.21
kazachstania_yasuniensis	kazachstania_yasuniensis	#N/A	Kazachstania_yasuniensis	yHMPu5000034708_kazachstania_yasuniensis_180604	g001209.m1	#N/A	Kazachstania	,	39.26319	,	4.0804			39263.19
Kazachstania_barnettii	Kazachstania_barnettii	#N/A	Kazachstania_barnettii	yHAB162_Kazachstania_barnettii_SPADES	g001263.m1	#N/A	Kazachstania	,	43.36564	,	4.0478			43365.64
Kazachstania_psychrophila	Kazachstania_psychrophila	#N/A	Kazachstania_psychrophila	yHMPu5000034706_Kazachstania_psychrophila_SPADES	g002942.m1	#N/A	Kazachstania	,	41.18863	,	4.0893			41188.63
Kazachstania_jinghongensis	Kazachstania_jinghongensis	#N/A	Kazachstania_jinghongensis	yHMPu5000037210_Kazachstania_jinghongensis_SPADES	g001801.m1	#N/A	Kazachstania	,	48.51643	,	5.2415			48516.43
kazachstaniasiamensis	kazachstania_siamensis	augustus_masked-NODE_61_length_63804_cov_14.6622_ID_121-processed-gene-0.80	Kazachstania_siamensis	yHAB143_kazachstania_siamensis_160519	g004344.m1	#N/A	Kazachstania	,	32.30443	,	4.0753			32304.43
kazachstaniataianensis	kazachstania_taianensis	augustus_masked-NODE_5_length_452406_cov_49.1731_ID_9-processed-gene-3.120	Kazachstania_taianensis	yHAB147_kazachstania_taianensis_160519	g004007.m1	#N/A	Kazachstania	,	26.77711	,	4.3032			26777.11
kazachstanianaganishii	kazachstania_naganishii	snap_masked-HE978326-processed-gene-0.118	Kazachstania_naganishii	kazachstania_naganishii	g001440.m1	XP_022467125.1	Kazachstania	,	31.97654	,	4.4904			31976.54
kazachstaniamartiniae	kazachstania_martiniae	snap_masked-scf7180000030514-processed-gene-0.103	Kazachstania_martiniae	yHAB132_kazachstania_martiniae_160519	g004439.m1	#N/A	Kazachstania	,	30.1896	,	4.3839			30189.6
kazachstaniaturicensis	kazachstania_turicensis	snap_masked-scf7180000049581-processed-gene-4.63	Kazachstania_turicensis	yHMPu5000040961_kazachstania_turicensis_201018	g004713.m1	#N/A	Kazachstania	,	48.82722	,	4.3272			48827.22
kazachstaniakunashirensis	kazachstania_kunashirensis	snap_masked-scf7180000008985-processed-gene-1.61	Kazachstania_kunashirensis	yHAB160_kazachstania_kunashirensis_160519	g005050.m1	#N/A	Kazachstania	,	43.41379	,	4.328			43413.79
kazachstaniaspencerorum	kazachstania_spencerorum	snap_masked-scf7180000055600-processed-gene-0.48	Kazachstania_spencerorum	yHAB155_kazachstania_spencerorum_160519	g002874.m1	#N/A	Kazachstania	,	46.07466	,	4.6212			46074.66
kazachstaniaafricana	kazachstania_africana	augustus_masked-scaffold_2-processed-gene-13.205	Kazachstania_africana	yHAB137_kazachstania_africana_160519	g003755.m1	XP_003956120.1	Kazachstania	,	40.02251	,	9.5003			40022.51
kazachstaniaviticola	kazachstania_viticola	snap_masked-scf7180000050361-processed-gene-0.67	Kazachstania_viticola	yHAB158_kazachstania_viticola_160519.haplomerger2	g001183.m1	#N/A	Kazachstania	,	40.5042	,	4.8594			40504.2
naumovozymadairenensis	naumovozyma_dairenensis	augustus_masked-NC_016482.1-processed-gene-7.22	Naumovozyma_dairenensis	yHMPu5000034872_naumovozyma_dairenensis_180604	g002752.m1	XP_003669889.1	Naumovozyma	,	50.83124	,	4.1573			50831.24
naumovozymacastellii	naumovozyma_castellii	snap_masked-HE576752-processed-gene-12.71	Naumovozyma_castellii	yHMPu5000034871_naumovozyma_castellii_180604	g001098.m1	#N/A	Naumovozyma	,	40.86545	,	4.3861			40865.45
naumovozyma_baii	naumovozyma_baii	#N/A	Naumovozyma_baii	yHMPu5000034894_naumovozyma_baii_190924	g000794.m1	#N/A	Naumovozyma	,	37.34486	,	5.8373			37344.86
tetrapisisporairiomotensis	tetrapisispora_iriomotensis	augustus_masked-flattened_line_57-processed-gene-0.130	Tetrapisispora_iriomotensis	yHMPu5000034876_Tetrapisispora_iriomotensis_SPADES	g003416.m1	#N/A	Tetrapisispora	,	41.90352	,	4.3115			41903.52
tetrapisisporanamnaonensis	tetrapisispora_namnaonensis	#N/A	Tetrapisispora_namnaoensis	yHMPu5000034877_tetrapisispora_namnaonensis_160519	g001993.m1	#N/A	Tetrapisispora	,	35.97886	,	4.1963			35978.86
tetrapisisporablattae	tetrapisispora_blattae	snap_masked-NC_020185-processed-gene-25.62	Tetrapisispora_blattae	yHMPu5000034874_tetrapisispora_blattae_190924	g003018.m1	XP_004178357.1	Tetrapisispora	,	52.65595	,	4.1007			52655.95
vanderwaltozymapolyspora	vanderwaltozyma_polyspora	snap_masked-NW_001834637.1-processed-gene-0.82	Vanderwaltozyma_polyspora	yHMPu5000034869_vanderwaltozyma_polyspora_180604	g005365.m1	XP_001643905.1	Vanderwaltozyma	,	46.01007	,	4.2926			46010.07
Vanderwaltozyma_yarrowii	Vanderwaltozyma_yarrowii	#N/A	Vanderwaltozyma_yarrowii	yHMPu5000034868_Vanderwaltozyma_yarrowii_S33_SPADES	g001449.m1	#N/A	Vanderwaltozyma	,	44.66004	,	3.9007			44660.04
vanderwaltozyma_tropicalis	vanderwaltozyma_tropicalis	#N/A	Vanderwaltozyma_tropicalis	yHMPu5000026257_vanderwaltozyma_tropicalis_190924	g003717.m1	#N/A	Vanderwaltozyma	,	34.86475	,	4.6183			34864.75
Vanderwaltozyma_verrucispora	Vanderwaltozyma_verrucispora	#N/A	Vanderwaltozyma_verrucispora	yHMPu5000037837_Vanderwaltozyma_verrucispora_S176_SPADES	g005143.m1	#N/A	Vanderwaltozyma	,	41.38653	,	4.6518			41386.53
torulasporapretoriensis	torulaspora_pretoriensis	augustus_masked-flattened_line_3-processed-gene-4.12	Torulaspora_pretoriensis	yHMPu5000034881_Torulaspora_pretoriensis_SPADES	g004938.m1	#N/A	Torulaspora	,	35.57586	,	4.9184			35575.86
torulasporamicroellipsoides	torulaspora_microellipsoides	augustus_masked-flattened_line_52-processed-gene-0.132	Torulaspora_microellipsoides	yHMPu5000035651_Torulaspora_microellipsoides_SPADES	g002639.m1	#N/A	Torulaspora	,	38.43885	,	4.9983			38438.85
torulasporafranciscae	torulaspora_franciscae	snap_masked-flattened_line_10-processed-gene-1.40	Torulaspora_franciscae	yHMPu5000026152_Torulaspora_franciscae_SPADES	g004752.m1	#N/A	Torulaspora	,	35.29763	,	4.7552			35297.63
torulasporadelbrueckii	torulaspora_delbrueckii	snap_masked-scaffold_4-processed-gene-1.34	Torulaspora_delbrueckii	yHMPu5000035653_torulaspora_delbrueckii_160613	g001776.m1	XP_003680850.1	Torulaspora	,	35.87571	,	5.2494			35875.71
torulaspora_globosa	torulaspora_globosa	#N/A	Torulaspora_globosa	yHMPu5000034880_torulaspora_globosa_160519	g001826.m1	#N/A	Torulaspora	,	32.85382	,	4.2401			32853.82
torulaspora_maleeae	torulaspora_maleeae	snap_masked-NODE_5_length_764704_cov_59.617_ID_1253-processed-gene-6.109	Torulaspora_maleeae	yHMPu5000035652_torulaspora_maleeae_160613	g003867.m1	#N/A	Torulaspora	,	33.46367	,	4.5261			33463.67
zygosaccharomyces_bisporus	zygosaccharomyces_bisporus	snap_masked-flattened_line_16-processed-gene-0.27	Zygosaccharomyces_bisporus	yHMPu5000034866_zygosaccharomyces_bisporus_160519	g005101.m1	#N/A	Zygosaccharomyces	,	30.78561	,	4.4988			30785.61
zygosaccharomyces_gambellarensis	zygosaccharomyces_gambellarensis	#N/A	Zygosaccharomyces_gambellarensis	yHDO565_zygosaccharomyces_gambellarensis_180604	g005022.m1	#N/A	Zygosaccharomyces	,	33.46945	,	4.6711			33469.45
zygosaccharomycesbailii	Zygosaccharomyces_bailii	augustus_masked-ZYBA0S10-processed-gene-3.53	Zygosaccharomyces_bailii	zygosaccharomyces_bailii	g001048.m1	CDF91308.1	Zygosaccharomyces	,	31.26516	,	4.8738			31265.16
zygosaccharomyceskombuchaensis	Zygosaccharomyces_kombuchaensis	snap_masked-NODE_1_length_271579_cov_10.1807_ID_1-processed-gene-1.23	Zygosaccharomyces_kombuchaensis	yHMPu5000034865_zygosaccharomyces_kombuchaensis_160519	g000063.m1	#N/A	Zygosaccharomyces	,	30.40245	,	4.6402			30402.45
zygosaccharomyces_lentus	zygosaccharomyces_lentus	#N/A	Zygosaccharomyces_lentus	yHMPu5000034864_zygosaccharomyces_lentus_170307	g004388.m1	#N/A	Zygosaccharomyces	,	30.25931	,	5.4041			30259.31
zygosaccharomyces_mellis	zygosaccharomyces_mellis	#N/A	Zygosaccharomyces_mellis	yHDO572_zygosaccharomyces_mellis_180604	g002135.m1	#N/A	Zygosaccharomyces	,	32.94388	,	5.2563			32943.88
zygosaccharomyces_pseudorouxii	zygosaccharomyces_pseudorouxii	#N/A	Zygosaccharomyces_pseudorouxii	yHMPu5000037836_zygosaccharomyces_pseudorouxii_201018.haplomerger2	g004410.m1	#N/A	Zygosaccharomyces	,	32.81375	,	4.7807			32813.75
zygosaccharomyces_siamensis	zygosaccharomyces_siamensis	#N/A	Zygosaccharomyces_siamensis	yHMPu5000035626_zygosaccharomyces_siamensis_190924	g000372.m1	#N/A	Zygosaccharomyces	,	32.81682	,	5.4515			32816.82

Species Name	seqID	HMMProfileID	Y1000_TreeID	Y1000_DatasetID	Y1000_protID	ncbiID	group	,	Molecular Weight (KDa)	,	Isoelectric Point			Molecular Weight (Da)
zygotorulasporaflorentina	zygotorulaspora_florentina	augustus_masked-NODE_6_length_562868_cov_24.3835_ID_11-processed-gene-2.47	Zygotorulaspora_florentina	yHMPu5000034862_zygotorulaspora_florentina_160519	g004327.m1	#N/A	Zygotorulaspora	,	37.61049	,	6.2991			37610.49
zygotorulasporamrakii	zygotorulaspora_mrakii	snap_masked-NODE_2_length_708340_cov_18.0358_ID_2767-processed-gene-4.105	Zygotorulaspora_mrakii	yHMPu5000026256_zygotorulaspora_mrakii_170307	g002003.m1	#N/A	Zygotorulaspora	,	39.02446	,	5.0952			39024.46
zygotorulaspora_chibaensis	zygotorulaspora_chibaensis	#N/A	Zygotorulaspora_chibaensis	yHMPu5000037204_zygotorulaspora_chibaensis_210210	g002973.m1	#N/A	Zygotorulaspora	,	37.70335	,	4.9361			37703.35
zygotorulaspora_danielsina	zygotorulaspora_danielsina	#N/A	Zygotorulaspora_danielsina	yHMPu5000037205_zygotorulaspora_danielsina_210210	g002121.m1	#N/A	Zygotorulaspora	,	36.47326	,	5.7074			36473.26
zygotorulaspora_mrakii	zygotorulaspora_mrakii	snap_masked-NODE_2_length_708340_cov_18.0358_ID_2767-processed-gene-4.105	Zygotorulaspora_mrakii	yHMPu5000026256_zygotorulaspora_mrakii_170307	g002003.m1	#N/A	Zygotorulaspora	,	39.02446	,	5.0952			39024.46
hagleromyces_aurorensis	hagleromyces_aurorensis	#N/A	Hagleromyces_aurorensis	yHDO579_hagleromyces_aurorensis_180604	g002965.m1	#N/A	Zygotorulaspora	,	53.61132	,	4.3924			53611.32
lachanceakluyveri	lachancea_kluyveri	snap_masked-SAKL0G-processed-gene-15.78	Lachancea_kluyveri	yHMPu5000034694_lachancea_kluyveri_180604	g001117.m1	#N/A	Lachancea	,	34.00936	,	5.1086			34009.36
lachancea_mirantina	lachancea_mirantina	augustus_masked-LAMI0C-processed-gene-0.22	Lachancea_mirantina	yHMPu5000034691_lachancea_mirantina_180604	g001548.m1	SCU82809.1	Lachancea	,	33.72982	,	5.6031			33729.82
Lachancea_nothofagi	Lachancea_nothofagi	snap_masked-LANO0B-processed-gene-1.74	Lachancea_nothofagi	yHMPu5000034690_lachancea_nothofagi_180604	g001406.m1	SCU80770.1	Lachancea	,	38.10047	,	8.1531			38100.47
lachanceafantastica	lachancea_fantastica	#N/A	Lachancea_fantastica_nom._nud.	lachancea_fantastica	g003435.m1	SCU92434.1	Lachancea	,	37.40871	,	6.7695			37408.71
lachancealanzarotensis	lachancea_lanzarotensis	augustus_masked-LN736361.1-processed-gene-3.12	Lachancea_lanzarotensis	yHMPu5000034693_Lachancea_lanzarotensis_SPADES	g004947.m1	XP_022627210.1	Lachancea	,	37.58671	,	6.775			37586.71
lachanceawaltii	lachancea_waltii	snap_masked-AADM01000119.1-processed-gene-0.11	Lachancea_waltii	yHMPu5000026219_lachancea_waltii_180604	g003199.m1	#N/A	Lachancea	,	33.30709	,	9.1367			33307.09
lachanceaquebecensis	lachancea_quebecensis	snap_masked-LAQU0S22-processed-gene-0.116	Lachancea_quebecensis	yHMPu5000040963_lachancea_quebecuensis_200128	g002616.m1	#N/A	Lachancea	,	36.10041	,	8.3847			36100.41
lachanceathermotolerans	lachancea_thermotolerans	snap_masked-CU928168-processed-gene-14.65	Lachancea_thermotolerans	yHMPu5000034678_lachancea_thermotolerans_180604	g003213.m1	XP_002553478.1	Lachancea	,	32.42788	,	8.1613			32427.88
lachanceameyersii	lachancea_meyersii	snap_masked-LAME0G-processed-gene-17.24	Lachancea_meyersii	yHMPu5000034692_lachancea_meyersii_180604	g001214.m1	SCV01664.1	Lachancea	,	38.05227	,	9.1029			38052.27
lachanceadasiensis	lachancea_dasiensis	augustus_masked-LADA0A-processed-gene-7.6	Lachancea_dasiensis	yHMPu5000034697_lachancea_dasiensis_190924	g003259.m1	SCU78828.1	Lachancea	,	40.14557	,	7.4365			40145.57
lachanceafermentati	lachancea_fermentati	augustus_masked-LAFE0A-processed-gene-1.131	Lachancea_fermentati	yHMPu5000034695_lachancea_fermentati_201018	g004518.m1	SCV99344.1	Lachancea	,	31.72441	,	5.0251			31724.41
eremotheciugossypii	eremotheciu_gossypii	#N/A	Eremothecium_gossypii	eremothecium_gossypii	NA	NP_982880.2	Eremothecium	,	25.22526	,	10.297			25225.26
eremotheciumcymbalariae	eremothecium_cymbalariae	snap_masked-NC_016453.1-processed-gene-3.121	Eremothecium_cymbalariae	eremothecium_cymbalariae	g002832.m1	XP_003646754.1	Eremothecium	,	32.10557	,	4.7546			32105.57
eremotheciumcoryli	eremothecium_coryli	maker-AZAH01000009.1-snap-gene-0.44	Eremothecium_coryli	eremothecium_coryli	g004362.m1	#N/A	Eremothecium	,	27.77677	,	8.5921			27776.77
eremotheciumsinecaudum	eremothecium_sinecaudum	#N/A	Eremothecium_sinecaudum	eremothecium_sinecaudum	g003584.m1	XP_017989102.1	Eremothecium	,	32.91456	,	9.7132			32914.56
kluyveromyceslactis	kluyveromyces_lactis	#N/A	Kluyveromyces_lactis_CBS_2359	kluyveromyces_lactis	g000677.m1	XP_451721.1	Kluyveromyces	,	31.42798	,	5.2075			31427.98
kluyveromycesdobzhanskii	kluyveromyces_dobzhanskii	snap_masked-NODE_13_length_251415_cov_20.6991_ID_25-processed-gene-1.144	Kluyveromyces_dobzhanskii	yHMPu5000034710_kluyveromyces_dobzhanskii_160519	g001256.m1	CDO92201.1	Kluyveromyces	,	32.31271	,	7.0271			32312.71
kluyveromycesaestuarii	kluyveromyces_aestuarii	#N/A	Kluyveromyces_aestuarii_ATCC_18862	kluyveromyces_aestuarii	g000823.m1	#N/A	Kluyveromyces	,	30.65803	,	7.3002			30658.03
kluyveromyces_nonfermentans	kluyveromyces_nonfermentans	#N/A	Kluyveromyces_nonfermentans	yHMPu5000034701_kluyveromyces_nonfermentans_170307	g001343.m1	#N/A	Kluyveromyces	,	31.92737	,	8.7328			31927.37
kluyveromyces_siamensis	kluyveromyces_siamensis	#N/A	Kluyveromyces_siamensis	yHMPu5000034700_kluyveromyces_siamensis_180604	g003460.m1	#N/A	Kluyveromyces	,	30.40989	,	6.1228			30409.89
kluyveromyces_starmeri	kluyveromyces_starmeri	#N/A	Kluyveromyces_starmeri	yHDO570_kluyveromyces_starmeri_180604	g003161.m1	#N/A	Kluyveromyces	,	30.73575	,	6.0696			30735.75
kluyveromyces_wickerhamii	kluyveromyces_wickerhamii	#N/A	Kluyveromyces_wickerhamii	yHMPu5000034699_kluyveromyces_wickerhamii_170307	g000532.m1	#N/A	Kluyveromyces	,	33.53587	,	6.6051			33535.87
hanseniasporauvarum	hanseniaspora_uvarum	#N/A	Hanseniaspora_uvarum_DSMZ_2768	hanseniaspora_uvarum	g000873.m1	KKA03541.1	Hanseniaspora	,	44.31316	,	4.4615			44313.16
hanseniasporapseudoguilliermondi	hanseniaspora_pseudoguilliermondi	#N/A	Hanseniaspora_pseudoguilliermondii	yHMPu5000035695_hanseniaspora_pseudoguilliermondii_160519.haplomerger2	g000765.m1	#N/A	Hanseniaspora	,	42.93928	,	4.1968			42939.28
hanseniasporaclermontiae	hanseniaspora_clermontiae	#N/A	Hanseniaspora_clermontiae	yHMPu5000034963_hanseniaspora_clermontiae_160519.haplomerger2	g003387.m1	#N/A	Hanseniaspora	,	43.04594	,	4.4959			43045.94
hanseniasporavalbyensis	hanseniaspora_valbyensis	#N/A	Hanseniaspora_valbyensis	yHMPu5000034955_hanseniaspora_valbyensis_201018.haplomerger2	g000788.m1	OBA26588.1	Hanseniaspora	,	46.0101	,	4.4316			46010.1
hanseniasporavinae	hanseniaspora_vinae	#N/A	Hanseniaspora_vineae	yHMPu5000026147_hanseniaspora_vineae_190924.haplomerger2	g000163.m1	#N/A	Hanseniaspora	,	55.14142	,	4.9736			55141.42
cyberlindnerasuaveolens	cyberlindnera_suaveolens	#N/A	Cyberlindnera_suaveolens	yHMPu5000035687_cyberlindnera_suaveolens_160613	g005674.m1	#N/A	Cyberlindnera	,	37.19332	,	4.377			37193.32
cyberlindnerasaturnus	cyberlindnera_saturnus	#N/A	Cyberlindnera_saturnus	yHMPu5000035686_Cyberlindnera_saturnus_SPADES	g001009.m1	#N/A	Cyberlindnera	,	37.03333	,	4.3708			37033.33
cyberlindneramisumaiensis	cyberlindnera_misumaiensis	#N/A	Cyberlindnera_misumaiensis	yHMPu5000034979_cyberlindnera_misumaiensis_160519	g002417.m1	#N/A	Cyberlindnera	,	30.88568	,	4.8579			30885.68
Cyberlindnerajadinii	Cyberlindnera_jadinii	#N/A	Cyberlindnera_jadinii	yHMPu5000035336_cyberlindnera_jadinii_180604.haplomerger2	g002673.m1	XP_020072378.1	Cyberlindnera	,	42.74321	,	4.5317			42743.21
Cyberlindnerafabianii	Cyberlindnera_fabianii	#N/A	Cyberlindnera_fabianii	yHMPu5000035701_cyberlindnera_fabianii_160519	g005778.m1	ONH68477.1	Cyberlindnera	,	39.35018	,	6.1188			39350.18
cyberlindneramaclurae	cyberlindnera_maclurae	#N/A	Cyberlindnera_maclurae	yHMPu5000035699_cyberlindnera_maclurae_160613	g003717.m1	#N/A	Cyberlindnera	,	23.21122	,	4.8781			23211.22
wickerhamomycesalni	wickerhamomyce_salni	#N/A	Wickerhamomyces_alni	yHMPu5000035274_wickerhamomyces_alni_170307	g001244.m1	#N/A	Wickerhamomyces	,	39.20614	,	5.0895			39206.14
wickerhamomycescanadensis	wickerhamomyces_canadensis	#N/A	Wickerhamomyces_canadensis	yHMPu5000035639_wickerhamomyces_canadensis_160613	g004559.m1	#N/A	Wickerhamomyces	,	25.01526	,	4.2917			25015.26
wickerhamomycesciferrii	wickerhamomyces_ciferrii	#N/A	Wickerhamomyces_ciferrii	yHMPu5000035269_wickerhamomyces_ciferrii_170307	g005482.m1,g003054.m1	XP_011277532.1	Wickerhamomyces	,	43.91976	,	4.1975			43919.76
Wickerhamomyceschambardii	Wickerhamomyces_chambardii	#N/A	Wickerhamomyces_chambardii	yHMPu5000035270_wickerhamomyces_chambardii_180604.haplomerger2	g001027.m1	#N/A	Wickerhamomyces	,	57.58908	,	4.48			57589.08
Wickerhamomyceshampshirensis	Wickerhamomyces_hampshirensis	#N/A	Wickerhamomyces_hampshirensis	yHMPu5000035268_wickerhamomyces_hampshirensis_160928	g002138.m1	#N/A	Wickerhamomyces	,	47.13197	,	4.7469			47131.97
Wickerhamomycesmucosus	Wickerhamomyces_mucosus	#N/A	Wickerhamomyces_mucosus	yHMPu5000038048_wickerhamomyces_mucosus_170912.haplomerger2	g002404.m1	#N/A	Wickerhamomyces	,	45.13403	,	4.7526			45134.03
Wickerhamomycespiperi	Wickerhamomyces_piperi	#N/A	Wickerhamomyces_pijperi	yHMPu5000035254_wickerhamomyces_pijperi_160928.haplomerger2	g002584.m1	#N/A	Wickerhamomyces	,	32.50732	,	4.0717			32507.32
komagataellaphaffi	komagataella_phaffi	#N/A	Komagataella_phaffii_GS115	komagataella_phaffi	g002262.m1	XP_002491603.1	Komagataella	,	35.0953	,	4.4338			35095.3
komagataellapopuli	komagataella_populi	#N/A	Komagataella_populi	yHMPu5000026274_komagataella_populi_160519	g004657.m1	#N/A	Komagataella	,	35.2525	,	4.5982			35252.5
komagataellapastoris	komagataella_pastoris	#N/A	Komagataella_pastoris_ATCC_28485	komagataella_pastoris	g002679.m1	ANZ75330.1	Komagataella	,	35.8892	,	4.4453			35889.2
Komagataellakurtzmanii	Komagataella_kurtzmanii	#N/A	Komagataella_kurtzmanii	yHMPu5000035676_komagataella_kurtzmanii_160613	g004231.m1	#N/A	Komagataella	,	35.15743	,	4.4338			35157.43
Komatagataella	Komatagaella_mondaviorum	#N/A	Komagataella_mondaviorum	yHMPu5000037220_komagataella_mondaviorum_210210	g004487.m1	#N/A	Komagataella	,	35.24556	,	4.3928			35245.56
Kuraishia	Kuraishia_floccosa	#N/A	Kuraishia_floccosa	yHMPu5000034935_kuraishia_floccosa_210210	g004700.m1	#N/A	Kuraishia	,	48.45106	,	4.1091			48451.06
NA	Asbhya_aceri	#N/A	Ashbya_aceri	ashbya_aceri	NA	AGO10388.1	Eremothecium							
Wickerhamomyces_anomalus	Wickerhamomyces_anomalus	#N/A	Wickerhamomyces_anomalus	yHMPu5000035273_wickerhamomyces_anomalus_180604.haplomerger2	g001657.m1	TBD	Wickerhamomyces							

saccharomyces_mikatae.4711/1-325	saccharomyces_mikatae	4711/1-325	Saccharomyces_mikatae	0	0	1	*Saccharomyces_mikatae*
saccharomyces_paradoxus.4820/1-321	saccharomyces_paradoxus	4820/1-321	Saccharomyces_paradoxus	0	0	1	
Saccharomyces_cerevisiae.2608/1-321	Saccharomyces_cerevisiae	2608/1-321	Saccharomyces_cerevisiae	0	0	1	
saccharomyces_uvarum.2005/523-836	saccharomyces_uvarum	2005/523-836	Saccharomyces_uvarum	0	0	1	1
saccharomyces_eubayanus.4603/581-888	saccharomyces_eubayanus	4603/581-888	Saccharomyces_eubayanus	0	0	1	
saccharomyces_arboricola.2054/541-857	saccharomyces_arboricola	2054/541-857	Saccharomyces_arboricola	0	1	1	
torulaspora_delbrueckii.2077/1-320	torulaspora_delbrueckii	2077/1-320	Torulaspora_delbrueckii	0	0	1	
torulaspora_franciscae.1384/1-315	torulaspora_franciscae	1384/1-315	Torulaspora_franciscae	0	0	1	
torulaspora_pretoriensis.3518/1-316	torulaspora_pretoriensis	3518/1-316	Torulaspora_pretoriensis	0	0	1	
torulaspora_pretoriensis.3519/1-316	torulaspora_pretoriensis	3519/1-316	Torulaspora_pretoriensis	0	0	1	
torulaspora_microellipsoides.4259/1-344	torulaspora_microellipsoides	4259/1-344	Torulaspora_microellipsoides	0	0	1	
zygosaccharomyces_bailii.3872/1-282	zygosaccharomyces_bailii	3872/1-282	Zygosaccharomyces_bailii	0	0	1	
zygotorulaspora_florentina.4182/586-923	zygotorulaspora_florentina	4182/586-923	Zygotorulaspora_florentina	0	0	1	
zygosaccharomyces_kombuchaensis.1039/1-275	zygosaccharomyces_kombuchaensis	1039/1-275	Zygosaccharomyces_kombuchaensis	0	0	1	
zygosaccharomyces_bisporus.1211/1-279	zygosaccharomyces_bisporus	1211/1-279	Zygosaccharomyces_bisporus	0	0	1	
zygotorulaspora_mrakii.2527/1-350	zygotorulaspora_mrakii	2527/1-350	Zygotorulaspora_mrakii	0	0	2	
zygosaccharomyces_rouxii.874/1-294	zygosaccharomyces_rouxii	874/1-294	Zygosaccharomyces_rouxii	0	0	0	
torulaspora_maleeae.3383/1-304	torulaspora_maleeae	3383/1-304	Torulaspora_maleeae	0	0	1	
saccharomyces_kudriavzevii.4688/573-833	saccharomyces_kudriavzevii	4688/573-833	Saccharomyces_kudriavzevii	0	0	1	
lachancea_kluyveri.4105/1-299	lachancea_kluyveri	4105/1-299	Lachancea_kluyveri	0	0	1	
naumovozyma_dairenensis.2516/1-258	naumovozyma_dairenensis	2516/1-258	Naumovozyma_dairenensis	0	0	1	
naumovozyma_dairenensis.2516/421-457	naumovozyma_dairenensis	2516/421-457	Naumovozyma_dairenensis	0	0	1	
kazachstania_viticola.4751/1-360	kazachstania_viticola	4751/1-360	Kazachstania_viticola	0	0	1	
naumovozyma_castellii.578/448-789	naumovozyma_castellii	578/448-789	Naumovozyma_castellii	0	0	1	
lachancea_fermentati.50/7-173	lachancea_fermentati	50/7-173	Lachancea_fermentati	0	0	1	
lachancea_fermentati.50/195-281	lachancea_fermentati	50/195-281	Lachancea_fermentati	0	0	1	
lachancea_cidri.2690/37-204	lachancea_cidri	2690/37-204	Lachancea_cidri	0	0	0	
lachancea_cidri.2690/225-312	lachancea_cidri	2690/225-312	Lachancea_cidri	0	0	0	
nakaseomyces_delphensis.4813/1-136	nakaseomyces_delphensis	4813/1-136	Nakaseomyces_delphensis	0	0	1	
nakaseomyces_delphensis.4813/185-236	nakaseomyces_delphensis	4813/185-236	Nakaseomyces_delphensis	0	0	1	
vanderwaltozyma_polyspora.1882/5-40	vanderwaltozyma_polyspora	1882/5-40	Vanderwaltozyma_polyspora	0	0	1	
vanderwaltozyma_polyspora.1882/129-163	vanderwaltozyma_polyspora	1882/129-163	Vanderwaltozyma_polyspora	0	0	1	
vanderwaltozyma_polyspora.1882/269-407	vanderwaltozyma_polyspora	1882/269-407	Vanderwaltozyma_polyspora	0	0	1	
kluyveromyces_aestuarii.3100/2-165	kluyveromyces_aestuarii	3100/2-165	Kluyveromyces_aestuarii	0	0	0	
kluyveromyces_aestuarii.3100/171-271	kluyveromyces_aestuarii	3100/171-271	Kluyveromyces_aestuarii	0	0	0	
nakaseomyces_bacillisporus.3279/3-99	nakaseomyces_bacillisporus	3279/3-99	Nakaseomyces_bacillisporus	0	0	1	
nakaseomyces_bacillisporus.3279/192-272	nakaseomyces_bacillisporus	3279/192-272	Nakaseomyces_bacillisporus	0	0	1	
nakaseomyces_bracarensis.2954/1-119	nakaseomyces_bracarensis	2954/1-119	Nakaseomyces_bracarensis	0	0	1	
nakaseomyces_bracarensis.2954/121-270	nakaseomyces_bracarensis	2954/121-270	Nakaseomyces_bracarensis	0	0	1	
lachancea_mirantina.815/3-303	lachancea_mirantina	815/3-303	Lachancea_mirantina	0	0	1	
nakaseomyces_nivariensis.2803/1-131	nakaseomyces_nivariensis	2803/1-131	Nakaseomyces_nivariensis	0	0	1	
nakaseomyces_nivariensis.2803/113-233	nakaseomyces_nivariensis	2803/113-233	Nakaseomyces_nivariensis	0	0	1	
lachancea_quebecensis.4341/20-160	lachancea_quebecensis	4341/20-160	Lachancea_quebecensis	0	0	1	
lachancea_quebecensis.4341/175-262	lachancea_quebecensis	4341/175-262	Lachancea_quebecensis	0	0	1	
lachancea_waltii.2068/10-274	lachancea_waltii	2068/10-274	Lachancea_waltii	0	0	1	
candida_glabrata.4846/1-104	candida_glabrata	4846/1-104	Candida_glabrata	0	0	0	
candida_glabrata.4846/113-241	candida_glabrata	4846/113-241	Candida_glabrata	0	0	0	
kazachstania_bromeliacearum.151/1-128	kazachstania_bromeliacearum	151/1-128	Kazachstania_bromeliacearum	0	0	1	
kazachstania_bromeliacearum.151/123-235	kazachstania_bromeliacearum	151/123-235	Kazachstania_bromeliacearum	0	0	1	
nakaseomyces_castellii.4169/1-389	nakaseomyces_castellii	4169/1-389	Nakaseomyces_castellii	0	0	1	
kazachstania_turicensis.4764/1-137	kazachstania_turicensis	4764/1-137	Kazachstania_turicensis	0	0	1	
kazachstania_turicensis.4764/312-424	kazachstania_turicensis	4764/312-424	Kazachstania_turicensis	0	0	1	
kazachstania_naganishii.4691/6-132	kazachstania_naganishii	4691/6-132	Kazachstania_naganishii	0	0	1	
kazachstania_naganishii.4691/183-284	kazachstania_naganishii	4691/183-284	Kazachstania_naganishii	0	0	1	
lachancea_lanzarotensis.976/4-340	lachancea_lanzarotensis	976/4-340	Lachancea_lanzarotensis	0	0	1	
lachancea_dasiensis.337/5-360	lachancea_dasiensis	337/5-360	Lachancea_dasiensis	0	0	1	
kazachstania_martiniae.4455/1-41	kazachstania_martiniae	4455/1-41	Kazachstania_martiniae	0	0	1	
kazachstania_martiniae.4455/117-266	kazachstania_martiniae	4455/117-266	Kazachstania_martiniae	0	0	1	
lachancea_nothofagi.389/11-289	lachancea_nothofagi	389/11-289	Lachancea_nothofagi	0	0	1	
kazachstania_kunashirensis.4945/1-131	kazachstania_kunashirensis	4945/1-131	Kazachstania_kunashirensis	0	0	2	
kazachstania_kunashirensis.4945/219-375	kazachstania_kunashirensis	4945/219-375	Kazachstania_kunashirensis	0	0	2	
lachancea_fantastica.3409/4-159	lachancea_fantastica	3409/4-159	Lachancea_fantastica	0	0	0	
lachancea_fantastica.3409/290-341	lachancea_fantastica	3409/290-341	Lachancea_fantastica	0	0	0	
tetrapisispora_iriomotensis.4707/5-135	tetrapisispora_iriomotensis	4707/5-135	Tetrapisispora_iriomotensis	0	0	1	
tetrapisispora_iriomotensis.4707/184-374	tetrapisispora_iriomotensis	4707/184-374	Tetrapisispora_iriomotensis	0	0	1	
kazachstania_africana.2163/1-129	kazachstania_africana	2163/1-129	Kazachstania_africana	0	0	1	
kazachstania_africana.2163/200-352	kazachstania_africana	2163/200-352	Kazachstania_africana	0	0	1	
kluyveromyces_lactis.677/61-176	kluyveromyces_lactis	677/61-176	Kluyveromyces_lactis	0	0	0	
kluyveromyces_lactis.677/248-278	kluyveromyces_lactis	677/248-278	Kluyveromyces_lactis	0	0	0	
lachancea_meyersii.3908/11-299	lachancea_meyersii	3908/11-299	Lachancea_meyersii	0	0	1	
kazachstania_siamensis.3992/1-129	kazachstania_siamensis	3992/1-129	Kazachstania_siamensis	0	0	1	
kazachstania_siamensis.3992/152-287	kazachstania_siamensis	3992/152-287	Kazachstania_siamensis	0	0	1	
kluyveromyces_dobzhanskii.483/44-194	kluyveromyces_dobzhanskii	483/44-194	Kluyveromyces_dobzhanskii	0	0	1	
kluyveromyces_dobzhanskii.483/236-281	kluyveromyces_dobzhanskii	483/236-281	Kluyveromyces_dobzhanskii	0	0	1	
eremothecium_cymbalariae.2716/144-220	eremothecium_cymbalariae	2716/144-220	Eremothecium_cymbalariae	0	0	1	

eremothecium_cymbalariae.2716/259-284	eremothecium_cymbalariae	2716/259-284	Eremothecium_cymbalariae	0	0	1	
lachancea_thermotolerans.1896/10-57	lachancea_thermotolerans	1896/10-57	Lachancea_thermotolerans	0	0	1	
lachancea_thermotolerans.1896/87-183	lachancea_thermotolerans	1896/87-183	Lachancea_thermotolerans	0	0	1	
kazachstania_taianensis.3897/1-44	kazachstania_taianensis	3897/1-44	Kazachstania_taianensis	0	0	1	
kazachstania_taianensis.3897/130-238	kazachstania_taianensis	3897/130-238	Kazachstania_taianensis	0	0	1	
kazachstania_aerobia.2672/1-349	kazachstania_aerobia	2672/1-349	Kazachstania_aerobia	0	0	1	
kazachstania_unispora.3458/1-342	kazachstania_unispora	3458/1-342	Kazachstania_unispora	0	0	1	
kazachstania_yakushimaensis.3740/307-438	kazachstania_yakushimaensis	3740/307-438	Kazachstania_yakushimaensis	0	0	0	
kazachstania_yakushimaensis.3740/716-872	kazachstania_yakushimaensis	3740/716-872	Kazachstania_yakushimaensis	0	0	0	
kazachstania_spencerorum.3580/3-278	kazachstania_spencerorum	3580/3-278	Kazachstania_spencerorum	0	0	1	
kazachstania_spencerorum.3580/351-408	kazachstania_spencerorum	3580/351-408	Kazachstania_spencerorum	0	0	1	
kazachstania_rosinii.1249/3-239	kazachstania_rosinii	1249/3-239	Kazachstania_rosinii	0	0	0	
kazachstania_solicola.5780/1-137	kazachstania_solicola	5780/1-137	Kazachstania_solicola	0	0	1	
kazachstania_solicola.5780/187-342	kazachstania_solicola	5780/187-342	Kazachstania_solicola	0	0	1	
tetrapisispora_blattae.1015/1-244	tetrapisispora_blattae	1015/1-244	Tetrapisispora_blattae	0	0	1	
tetrapisispora_blattae.1015/304-465	tetrapisispora_blattae	1015/304-465	Tetrapisispora_blattae	0	0	1	
tetrapisispora_namnaonensis.1469/6-127	tetrapisispora_namnaonensis	1469/6-127	Tetrapisispora_namnaonensis	0	0	0	
kluyveromyces_marxianus.2881/64-175	kluyveromyces_marxianus	2881/64-175	Kluyveromyces_marxianus	0	0	0	
eremothecium_coryli.3485/574-629	eremothecium_coryli	3485/574-629	Eremothecium_coryli	0	0	1	
lachancea_nothofagi.512/203-358	lachancea_nothofagi	512/203-358	Lachancea_nothofagi	0	0	1	

#=GS	saccharomyces_mikatae.4711/1-325	DE	[subseq	from]	snap_masked-Smik_8-processed-gene-4.133-mRNA-1_1	gene=snap_masked-Smik_8-processed-gene-4.133	CDS=1-975																	
#=GS	saccharomyces_paradoxus.4820/1-321	DE	[subseq	from]	genemark-Spar_8-processed-gene-4.108-mRNA-1_1	gene=genemark-Spar_8-processed-gene-4.108	CDS=1-963																	
#=GS	Saccharomyces_cerevisiae.2608/1-321	DE	[subseq	from]	YHR195W	NVJ1	SGDID:S000001238,	Chr	VIII	from	490747-491712,	Genome	Release	64-2-1,	Verified	ORF,	Nuclear envelope protein; anchored to the nuclear inner membrane, that interacts	during	piecemeal	microautophagy	of	the	nucleus	(PMN)"
#=GS	saccharomyces_uvarum.2005/523-836	DE	[subseq	from]	augustus_masked-Sbay_15-processed-gene-6.183-mRNA-1_1	gene=augustus_masked-Sbay_15-processed-gene-6.183	CDS=1-4203																	
#=GS	saccharomyces_eubayanus.4603/581-888	DE	[subseq	from]	augustus_masked-chrXV-processed-gene-6.90-mRNA-1_1	gene=augustus_masked-chrXV-processed-gene-6.90	CDS=1-4320																	
#=GS	saccharomyces_arboricola.2054/541-857	DE	[subseq	from]	augustus_masked-NC_026178.1-processed-gene-4.111-mRNA-1_1	gene=augustus_masked-NC_026178.1-processed-gene-4.111	CDS=1-2571																	
#=GS	torulaspora_delbrueckii.2077/1-320	DE	[subseq	from]	snap_masked-scaffold_4-processed-gene-1.34-mRNA-1_1	gene=snap_masked-scaffold_4-processed-gene-1.34	CDS=1-960																	
#=GS	torulaspora_franciscae.1384/1-315	DE	[subseq	from]	snap_masked-flattened_line_10-processed-gene-1.40-mRNA-1_1	gene=snap_masked-flattened_line_10-processed-gene-1.40	CDS=1-945																	
#=GS	torulaspora_pretoriensis.3518/1-316	DE	[subseq	from]	augustus_masked-flattened_line_3-processed-gene-4.12-mRNA-1_1	gene=augustus_masked-flattened_line_3-processed-gene-4.12	CDS=1-948																	
#=GS	torulaspora_pretoriensis.3519/1-316	DE	[subseq	from]	augustus_masked-flattened_line_3-processed-gene-4.38-mRNA-1_1	gene=augustus_masked-flattened_line_3-processed-gene-4.38	CDS=1-948																	
#=GS	torulaspora_microellipsoides.4259/1-344	DE	[subseq	from]	augustus_masked-flattened_line_52-processed-gene-0.132-mRNA-1_1	gene=augustus_masked-flattened_line_52-processed-gene-0.132	CDS=1-1032																	
#=GS	zygosaccharomyces_bailii.3872/1-282	DE	[subseq	from]	augustus_masked-ZYBA0S10-processed-gene-3.53-mRNA-1_1	gene=augustus_masked-ZYBA0S10-processed-gene-3.53	CDS=1-846																	
#=GS	zygotorulaspora_florentina.4182/586-923	DE	[subseq	from]	augustus_masked-NODE_6_length_562868_cov_24.3835_ID_11-processed-gene-2.47-mRNA-1_1	gene=augustus_masked-NODE_6_length_562868_cov_24.3835_ID_11-processed-gene-2.47	CDS=1-2769																	
#=GS	zygosaccharomyces_kombuchaensis.1039/1-275	DE	[subseq	from]	snap_masked-NODE_1_length_271579_cov_10.1807_ID_1-processed-gene-1.23-mRNA-1_1	gene=snap_masked-NODE_1_length_271579_cov_10.1807_ID_1-processed-gene-1.23	CDS=1-825																	
#=GS	zygosaccharomyces_bisporus.1211/1-279	DE	[subseq	from]	snap_masked-flattened_line_16-processed-gene-0.27-mRNA-1_1	gene=snap_masked-flattened_line_16-processed-gene-0.27	CDS=1-837																	
#=GS	zygotorulaspora_mrakii.2527/1-350	DE	[subseq	from]	snap_masked-NODE_2_length_708340_cov_18.0358_ID_2767-processed-gene-4.105-mRNA-1_1	gene=snap_masked-NODE_2_length_708340_cov_18.0358_ID_2767-processed-gene-4.105	CDS=1-1050																	
#=GS	zygosaccharomyces_rouxii.874/1-294	DE	[subseq	from]	snap_masked-Zyro0B-processed-gene-6.126-mRNA-1_1	gene=snap_masked-Zyro0B-processed-gene-6.126	CDS=1-882																	
#=GS	torulaspora_maleeae.3383/1-304	DE	[subseq	from]	snap_masked-NODE_5_length_764704_cov_59.617_ID_1253-processed-gene-6.109-mRNA-1_1	gene=snap_masked-NODE_5_length_764704_cov_59.617_ID_1253-processed-gene-6.109	CDS=1-912																	
#=GS	saccharomyces_kudriavzevii.4688/573-833	DE	[subseq	from]	augustus_masked-Skud_8-processed-gene-3.158-mRNA-1_1	gene=augustus_masked-Skud_8-processed-gene-3.158	CDS=1-6507																	
#=GS	lachancea_kluyveri.4105/1-299	DE	[subseq	from]	snap_masked-SAKL0G-processed-gene-15.78-mRNA-1_1	gene=snap_masked-SAKL0G-processed-gene-15.78	CDS=1-897																	
#=GS	naumovozyma_dairenensis.2516/1-258	DE	[subseq	from]	augustus_masked-NC_016482.1-processed-gene-7.22-mRNA-1_1	gene=augustus_masked-NC_016482.1-processed-gene-7.22	CDS=1-1371																	
#=GS	naumovozyma_dairenensis.2516/421-457	DE	[subseq	from]	augustus_masked-NC_016482.1-processed-gene-7.22-mRNA-1_1	gene=augustus_masked-NC_016482.1-processed-gene-7.22	CDS=1-1371																	
#=GS	kazachstania_viticola.4751/1-360	DE	[subseq	from]	snap_masked-scf7180000050361-processed-gene-0.67-mRNA-1_1	gene=snap_masked-scf7180000050361-processed-gene-0.67	CDS=1-1080																	
#=GS	naumovozyma_castellii.578/448-789	DE	[subseq	from]	snap_masked-HE576752-processed-gene-12.71-mRNA-1_1	gene=snap_masked-HE576752-processed-gene-12.71	CDS=1-2379																	
#=GS	lachancea_fermentati.50/7-173	DE	[subseq	from]	augustus_masked-LAFE0A-processed-gene-1.131-mRNA-1_1	gene=augustus_masked-LAFE0A-processed-gene-1.131	CDS=1-843																	
#=GS	lachancea_fermentati.50/195-281	DE	[subseq	from]	augustus_masked-LAFE0A-processed-gene-1.131-mRNA-1_1	gene=augustus_masked-LAFE0A-processed-gene-1.131	CDS=1-843																	
#=GS	lachancea_cidri.2690/37-204	DE	[subseq	from]	augustus_masked-LACI0G-processed-gene-1.33-mRNA-1_1	gene=augustus_masked-LACI0G-processed-gene-1.33	CDS=1-936																	
#=GS	lachancea_cidri.2690/225-312	DE	[subseq	from]	augustus_masked-LACI0G-processed-gene-1.33-mRNA-1_1	gene=augustus_masked-LACI0G-processed-gene-1.33	CDS=1-936																	
#=GS	nakaseomyces_delphensis.4813/1-136	DE	[subseq	from]	augustus_masked-NADE0S27-processed-gene-3.39-mRNA-1_1	gene=augustus_masked-NADE0S27-processed-gene-3.39	CDS=1-708																	
#=GS	nakaseomyces_delphensis.4813/185-236	DE	[subseq	from]	augustus_masked-NADE0S27-processed-gene-3.39-mRNA-1_1	gene=augustus_masked-NADE0S27-processed-gene-3.39	CDS=1-708																	
#=GS	vanderwaltozyma_polyspora.1882/5-40	DE	[subseq	from]	snap_masked-NW_001834637.1-processed-gene-0.82-mRNA-1_1	gene=snap_masked-NW_001834637.1-processed-gene-0.82	CDS=1-1221																	
#=GS	vanderwaltozyma_polyspora.1882/129-163	DE	[subseq	from]	snap_masked-NW_001834637.1-processed-gene-0.82-mRNA-1_1	gene=snap_masked-NW_001834637.1-processed-gene-0.82	CDS=1-1221																	
#=GS	vanderwaltozyma_polyspora.1882/269-407	DE	[subseq	from]	snap_masked-NW_001834637.1-processed-gene-0.82-mRNA-1_1	gene=snap_masked-NW_001834637.1-processed-gene-0.82	CDS=1-1221																	
#=GS	kluyveromyces_aestuarii.3100/2-165	DE	[subseq	from]	snap_masked-NODE_4_length_706832_cov_27.5555_ID_7-processed-gene-0.39-mRNA-1_1	gene=snap_masked-NODE_4_length_706832_cov_27.5555_ID_7-processed-gene-0.39	CDS=1-816																	
#=GS	kluyveromyces_aestuarii.3100/171-271	DE	[subseq	from]	snap_masked-NODE_4_length_706832_cov_27.5555_ID_7-processed-gene-0.39-mRNA-1_1	gene=snap_masked-NODE_4_length_706832_cov_27.5555_ID_7-processed-gene-0.39	CDS=1-816																	
#=GS	nakaseomyces_bacillisporus.3279/3-99	DE	[subseq	from]	augustus_masked-NABA0S29-processed-gene-0.108-mRNA-1_1	gene=augustus_masked-NABA0S29-processed-gene-0.108	CDS=1-816																	
#=GS	nakaseomyces_bacillisporus.3279/192-272	DE	[subseq	from]	augustus_masked-NABA0S29-processed-gene-0.108-mRNA-1_1	gene=augustus_masked-NABA0S29-processed-gene-0.108	CDS=1-816																	
#=GS	nakaseomyces_bracarensis.2954/1-119	DE	[subseq	from]	snap_masked-CABR0S29-processed-gene-6.0-mRNA-1_1	gene=snap_masked-CABR0S29-processed-gene-6.0	CDS=1-810																	
#=GS	nakaseomyces_bracarensis.2954/121-270	DE	[subseq	from]	snap_masked-CABR0S29-processed-gene-6.0-mRNA-1_1	gene=snap_masked-CABR0S29-processed-gene-6.0	CDS=1-810																	
#=GS	lachancea_mirantina.815/3-303	DE	[subseq	from]	augustus_masked-LAMI0C-processed-gene-0.22-mRNA-1_1	gene=augustus_masked-LAMI0C-processed-gene-0.22	CDS=1-909																	
#=GS	nakaseomyces_nivariensis.2803/1-131	DE	[subseq	from]	snap_masked-CANI0S17-processed-gene-4.132-mRNA-1_1	gene=snap_masked-CANI0S17-processed-gene-4.132	CDS=1-699																	
#=GS	nakaseomyces_nivariensis.2803/113-233	DE	[subseq	from]	snap_masked-CANI0S17-processed-gene-4.132-mRNA-1_1	gene=snap_masked-CANI0S17-processed-gene-4.132	CDS=1-699																	
#=GS	lachancea_quebecensis.4341/20-160	DE	[subseq	from]	snap_masked-LAQU0S22-processed-gene-0.116-mRNA-1_1	gene=snap_masked-LAQU0S22-processed-gene-0.116	CDS=1-786																	
#=GS	lachancea_quebecensis.4341/175-262	DE	[subseq	from]	snap_masked-LAQU0S22-processed-gene-0.116-mRNA-1_1	gene=snap_masked-LAQU0S22-processed-gene-0.116	CDS=1-786																	
#=GS	lachancea_waltii.2068/10-274	DE	[subseq	from]	snap_masked-AADM01000119.1-processed-gene-0.11-mRNA-1_1	gene=snap_masked-AADM01000119.1-processed-gene-0.11	CDS=1-822																	
#=GS	candida_glabrata.4846/1-104	DE	[subseq	from]	snap_masked-CR380959-processed-gene-7.12-mRNA-1_1	gene=snap_masked-CR380959-processed-gene-7.12	CDS=1-723																	
#=GS	candida_glabrata.4846/113-241	DE	[subseq	from]	snap_masked-CR380959-processed-gene-7.12-mRNA-1_1	gene=snap_masked-CR380959-processed-gene-7.12	CDS=1-723																	
#=GS	kazachstania_bromeliacearum.151/1-128	DE	[subseq	from]	snap_masked-NODE_10_length_278180_cov_22.5073_ID_19-processed-gene-1.127-mRNA-1_1	gene=snap_masked-NODE_10_length_278180_cov_22.5073_ID_19-processed-gene-1.127	CDS=1-711																	
#=GS	kazachstania_bromeliacearum.151/123-235	DE	[subseq	from]	snap_masked-NODE_10_length_278180_cov_22.5073_ID_19-processed-gene-1.127-mRNA-1_1	gene=snap_masked-NODE_10_length_278180_cov_22.5073_ID_19-processed-gene-1.127	CDS=1-711																	

#=GS	nakaseomyces_castellii.4169/1-389	DE	[subseq	from]	snap_masked-CACA0S33-processed-gene-9.107-mRNA-1_1	gene=snap_masked-CACA0S33-processed-gene-9.107	CDS=1-1167
#=GS	kazachstania_turicensis.4764/1-137	DE	[subseq	from]	snap_masked-scf7180000049581-processed-gene-4.63-mRNA-1_1	gene=snap_masked-scf7180000049581-processed-gene-4.63	CDS=1-1272
#=GS	kazachstania_turicensis.4764/312-424	DE	[subseq	from]	snap_masked-scf7180000049581-processed-gene-4.63-mRNA-1_1	gene=snap_masked-scf7180000049581-processed-gene-4.63	CDS=1-1272
#=GS	kazachstania_naganishii.4691/6-132	DE	[subseq	from]	snap_masked-HE978326-processed-gene-0.118-mRNA-1_1	gene=snap_masked-HE978326-processed-gene-0.118	CDS=1-852
#=GS	kazachstania_naganishii.4691/183-284	DE	[subseq	from]	snap_masked-HE978326-processed-gene-0.118-mRNA-1_1	gene=snap_masked-HE978326-processed-gene-0.118	CDS=1-852
#=GS	lachancea_lanzarotensis.976/4-340	DE	[subseq	from]	augustus_masked-LN736361.1-processed-gene-3.12-mRNA-1_1	gene=augustus_masked-LN736361.1-processed-gene-3.12	CDS=1-1020
#=GS	lachancea_dasiensis.337/5-360	DE	[subseq	from]	augustus_masked-LADA0A-processed-gene-7.6-mRNA-1_1	gene=augustus_masked-LADA0A-processed-gene-7.6	CDS=1-1080
#=GS	kazachstania_martiniae.4455/1-41	DE	[subseq	from]	snap_masked-scf7180000030514-processed-gene-0.103-mRNA-1_1	gene=snap_masked-scf7180000030514-processed-gene-0.103	CDS=1-798
#=GS	kazachstania_martiniae.4455/117-266	DE	[subseq	from]	snap_masked-scf7180000030514-processed-gene-0.103-mRNA-1_1	gene=snap_masked-scf7180000030514-processed-gene-0.103	CDS=1-798
#=GS	lachancea_nothofagi.389/11-289	DE	[subseq	from]	snap_masked-LANO0B-processed-gene-1.74-mRNA-1_1	gene=snap_masked-LANO0B-processed-gene-1.74	CDS=1-867
#=GS	kazachstania_kunashirensis.4945/1-131	DE	[subseq	from]	snap_masked-scf7180000008985-processed-gene-1.61-mRNA-1_1	gene=snap_masked-scf7180000008985-processed-gene-1.61	CDS=1-1125
#=GS	kazachstania_kunashirensis.4945/219-375	DE	[subseq	from]	snap_masked-scf7180000008985-processed-gene-1.61-mRNA-1_1	gene=snap_masked-scf7180000008985-processed-gene-1.61	CDS=1-1125
#=GS	lachancea_fantastica.3409/4-159	DE	[subseq	from]	snap_masked-LAFA0F-processed-gene-10.9-mRNA-1_1	gene=snap_masked-LAFA0F-processed-gene-10.9	CDS=1-1023
#=GS	lachancea_fantastica.3409/290-341	DE	[subseq	from]	snap_masked-LAFA0F-processed-gene-10.9-mRNA-1_1	gene=snap_masked-LAFA0F-processed-gene-10.9	CDS=1-1023
#=GS	tetrapisispora_iriomotensis.4707/5-135	DE	[subseq	from]	augustus_masked-flattened_line_57-processed-gene-0.130-mRNA-1_1	gene=augustus_masked-flattened_line_57-processed-gene-0.130	CDS=1-1122
#=GS	tetrapisispora_iriomotensis.4707/184-374	DE	[subseq	from]	augustus_masked-flattened_line_57-processed-gene-0.130-mRNA-1_1	gene=augustus_masked-flattened_line_57-processed-gene-0.130	CDS=1-1122
#=GS	kazachstania_africana.2163/1-129	DE	[subseq	from]	augustus_masked-scaffold_2-processed-gene-13.205-mRNA-1_1	gene=augustus_masked-scaffold_2-processed-gene-13.205	CDS=1-1059
#=GS	kazachstania_africana.2163/200-352	DE	[subseq	from]	augustus_masked-scaffold_2-processed-gene-13.205-mRNA-1_1	gene=augustus_masked-scaffold_2-processed-gene-13.205	CDS=1-1059
#=GS	kluyveromyces_lactis.677/61-176	DE	[subseq	from]	snap_masked-CR382122-processed-gene-3.49-mRNA-1_1	gene=snap_masked-CR382122-processed-gene-3.49	CDS=1-837
#=GS	kluyveromyces_lactis.677/248-278	DE	[subseq	from]	snap_masked-CR382122-processed-gene-3.49-mRNA-1_1	gene=snap_masked-CR382122-processed-gene-3.49	CDS=1-837
#=GS	lachancea_meyersii.3908/11-299	DE	[subseq	from]	snap_masked-LAME0G-processed-gene-17.24-mRNA-1_1	gene=snap_masked-LAME0G-processed-gene-17.24	CDS=1-897
#=GS	kazachstania_siamensis.3992/1-129	DE	[subseq	from]	augustus_masked-NODE_61_length_63804_cov_14.6622_ID_121-processed-gene-0.80-mRNA-1_1	gene=augustus_masked-NODE_61_length_63804_cov_14.6622_ID_121-processed-gene-0.80	CDS=1-867
#=GS	kazachstania_siamensis.3992/152-287	DE	[subseq	from]	augustus_masked-NODE_61_length_63804_cov_14.6622_ID_121-processed-gene-0.80-mRNA-1_1	gene=augustus_masked-NODE_61_length_63804_cov_14.6622_ID_121-processed-gene-0.80	CDS=1-867
#=GS	kluyveromyces_dobzhanskii.483/44-194	DE	[subseq	from]	snap_masked-NODE_13_length_251415_cov_20.6991_ID_25-processed-gene-1.144-mRNA-1_1	gene=snap_masked-NODE_13_length_251415_cov_20.6991_ID_25-processed-gene-1.144	CDS=1-846
#=GS	kluyveromyces_dobzhanskii.483/236-281	DE	[subseq	from]	snap_masked-NODE_13_length_251415_cov_20.6991_ID_25-processed-gene-1.144-mRNA-1_1	gene=snap_masked-NODE_13_length_251415_cov_20.6991_ID_25-processed-gene-1.144	CDS=1-846
#=GS	eremothecium_cymbalariae.2716/144-220	DE	[subseq	from]	snap_masked-NC_016453.1-processed-gene-3.121-mRNA-1_1	gene=snap_masked-NC_016453.1-processed-gene-3.121	CDS=1-852
#=GS	eremothecium_cymbalariae.2716/259-284	DE	[subseq	from]	snap_masked-NC_016453.1-processed-gene-3.121-mRNA-1_1	gene=snap_masked-NC_016453.1-processed-gene-3.121	CDS=1-852
#=GS	lachancea_thermotolerans.1896/10-57	DE	[subseq	from]	snap_masked-CU928168-processed-gene-14.65-mRNA-1_1	gene=snap_masked-CU928168-processed-gene-14.65	CDS=1-549
#=GS	lachancea_thermotolerans.1896/87-183	DE	[subseq	from]	snap_masked-CU928168-processed-gene-14.65-mRNA-1_1	gene=snap_masked-CU928168-processed-gene-14.65	CDS=1-549
#=GS	kazachstania_taianensis.3897/1-44	DE	[subseq	from]	augustus_masked-NODE_5_length_452406_cov_49.1731_ID_9-processed-gene-3.120-mRNA-1_1	gene=augustus_masked-NODE_5_length_452406_cov_49.1731_ID_9-processed-gene-3.120	CDS=1-717
#=GS	kazachstania_taianensis.3897/130-238	DE	[subseq	from]	augustus_masked-NODE_5_length_452406_cov_49.1731_ID_9-processed-gene-3.120-mRNA-1_1	gene=augustus_masked-NODE_5_length_452406_cov_49.1731_ID_9-processed-gene-3.120	CDS=1-717
#=GS	kazachstania_aerobia.2672/1-349	DE	[subseq	from]	augustus_masked-scf7180000020169-processed-gene-0.188-mRNA-1_1	gene=augustus_masked-scf7180000020169-processed-gene-0.188	CDS=1-1053
#=GS	kazachstania_unispora.3458/1-342	DE	[subseq	from]	snap_masked-NODE_47_length_78712_cov_50.1529_ID_93-processed-gene-0.49-mRNA-1_1	gene=snap_masked-NODE_47_length_78712_cov_50.1529_ID_93-processed-gene-0.49	CDS=1-1032
#=GS	kazachstania_yakushimaensis.3740/307-438	DE	[subseq	from]	genemark-scf7180000044634-processed-gene-0.102-mRNA-1_1	gene=genemark-scf7180000044634-processed-gene-0.102	CDS=1-2616
#=GS	kazachstania_yakushimaensis.3740/716-872	DE	[subseq	from]	genemark-scf7180000044634-processed-gene-0.102-mRNA-1_1	gene=genemark-scf7180000044634-processed-gene-0.102	CDS=1-2616
#=GS	kazachstania_spencerorum.3580/3-278	DE	[subseq	from]	snap_masked-scf7180000055600-processed-gene-0.48-mRNA-1_1	gene=snap_masked-scf7180000055600-processed-gene-0.48	CDS=1-1224
#=GS	kazachstania_spencerorum.3580/351-408	DE	[subseq	from]	snap_masked-scf7180000055600-processed-gene-0.48-mRNA-1_1	gene=snap_masked-scf7180000055600-processed-gene-0.48	CDS=1-1224
#=GS	kazachstania_rosinii.1249/3-239	DE	[subseq	from]	snap_masked-scf7180000099615-processed-gene-0.31-mRNA-1_1	gene=snap_masked-scf7180000099615-processed-gene-0.31	CDS=1-1491
#=GS	kazachstania_solicola.5780/1-137	DE	[subseq	from]	augustus_masked-scf7180000046332-processed-gene-1.206-mRNA-1_1	gene=augustus_masked-scf7180000046332-processed-gene-1.206	CDS=1-1032
#=GS	kazachstania_solicola.5780/187-342	DE	[subseq	from]	augustus_masked-scf7180000046332-processed-gene-1.206-mRNA-1_1	gene=augustus_masked-scf7180000046332-processed-gene-1.206	CDS=1-1032
#=GS	tetrapisispora_blattae.1015/1-244	DE	[subseq	from]	snap_masked-NC_020185-processed-gene-25.62-mRNA-1_1	gene=snap_masked-NC_020185-processed-gene-25.62	CDS=1-1446
#=GS	tetrapisispora_blattae.1015/304-465	DE	[subseq	from]	snap_masked-NC_020185-processed-gene-25.62-mRNA-1_1	gene=snap_masked-NC_020185-processed-gene-25.62	CDS=1-1446
#=GS	tetrapisispora_namnaonensis.1469/6-127	DE	[subseq	from]	augustus_masked-NODE_17_length_366881_cov_78.9049_ID_33-processed-gene-2.114-mRNA-1_1	gene=augustus_masked-NODE_17_length_366881_cov_78.9049_ID_33-processed-gene-2.114	CDS=1-996
#=GS	kluyveromyces_marxianus.2881/64-175	DE	[subseq	from]	snap_masked-BBIL01000008.1-processed-gene-11.40-mRNA-1_1	gene=snap_masked-BBIL01000008.1-processed-gene-11.40	CDS=1-879
#=GS	eremothecium_coryli.3485/574-629	DE	[subseq	from]	maker-AZAH01000009.1-snap-gene-0.44-mRNA-1_1	gene=maker-AZAH01000009.1-snap-gene-0.44	CDS=1-2259
#=GS	lachancea_nothofagi.512/203-358	DE	[subseq	from]	snap_masked-LANO0B-processed-gene-3.66-mRNA-1_1	gene=snap_masked-LANO0B-processed-gene-3.66	CDS=1-1185

Sequences	HMMProfileID
Saccharomyces_mikatae	snap_masked-Smik_8-processed-gene-4.133
Saccharomyces_paradoxus	genemark-Spar_8-processed-gene-4.108
Saccharomyces_cerevisiae	YHR195W
Saccharomyces_uvarum	augustus_masked-Sbay_15-processed-gene-6.183
Saccharomyces_eubayanus	augustus_masked-chrXV-processed-gene-6.90
Saccharomyces_arboricola	augustus_masked-NC_026178.1-processed-gene-4.111
Torulaspora_delbrueckii	snap_masked-scaffold_4-processed-gene-1.34
Torulaspora_franciscae	snap_masked-flattened_line_10-processed-gene-1.40
Torulaspora_pretoriensis	augustus_masked-flattened_line_3-processed-gene-4.12
Torulaspora_pretoriensis	augustus_masked-flattened_line_3-processed-gene-4.38
Torulaspora_microellipsoides	augustus_masked-flattened_line_52-processed-gene-0.132
Zygosaccharomyces_bailii	augustus_masked-ZYBA0S10-processed-gene-3.53
Zygotorulaspora_florentina	augustus_masked-NODE_6_length_562868_cov_24.3835_ID_11-processed-gene-2.47
Zygosaccharomyces_kombuchaensis	snap_masked-NODE_1_length_271579_cov_10.1807_ID_1-processed-gene-1.23
Zygosaccharomyces_bisporus	snap_masked-flattened_line_16-processed-gene-0.27
Zygotorulaspora_mrakii	snap_masked-NODE_2_length_708340_cov_18.0358_ID_2767-processed-gene-4.105
Zygosaccharomyces_rouxii	snap_masked-Zyro0B-processed-gene-6.126
Torulaspora_maleeae	snap_masked-NODE_5_length_764704_cov_59.617_ID_1253-processed-gene-6.109
Saccharomyces_kudriavzevii	augustus_masked-Skud_8-processed-gene-3.158
Lachancea_kluyveri	snap_masked-SAKL0G-processed-gene-15.78
Naumovozyma_dairenensis	augustus_masked-NC_016482.1-processed-gene-7.22
Naumovozyma_dairenensis	augustus_masked-NC_016482.1-processed-gene-7.22
Kazachstania_viticola	snap_masked-scf7180000050361-processed-gene-0.67
Naumovozyma_castellii	snap_masked-HE576752-processed-gene-12.71
Lachancea_fermentati	augustus_masked-LAFE0A-processed-gene-1.131
Lachancea_cidri	augustus_masked-LACI0G-processed-gene-1.33
Nakaseomyces_delphensis	augustus_masked-NADE0S27-processed-gene-3.39
Vanderwaltozyma_polyspora	snap_masked-NW_001834637.1-processed-gene-0.82
Kluyveromyces_aestuarii	snap_masked-NODE_4_length_706832_cov_27.5555_ID_7-processed-gene-0.39
Nakaseomyces_bacillisporus	augustus_masked-NABA0S29-processed-gene-0.108
Nakaseomyces_bracarensis	snap_masked-CABR0S29-processed-gene-6.0
Lachancea_mirantina	augustus_masked-LAMI0C-processed-gene-0.22
Nakaseomyces_nivariensis	snap_masked-CANI0S17-processed-gene-4.132
Lachancea_quebecensis	snap_masked-LAQU0S22-processed-gene-0.116
Lachancea_waltii	snap_masked-AADM01000119.1-processed-gene-0.11
Candida_glabrata	snap_masked-CR380959-processed-gene-7.12
Kazachstania_bromeliacearum	snap_masked-NODE_10_length_278180_cov_22.5073_ID_19-processed-gene-1.127
Nakaseomyces_castellii	snap_masked-CACA0S33-processed-gene-9.107
Kazachstania_turicensis	snap_masked-scf7180000049581-processed-gene-4.63
Kazachstania_naganishii	snap_masked-HE978326-processed-gene-0.118
Lachancea_lanzarotensis	augustus_masked-LN736361.1-processed-gene-3.12
Lachancea_dasiensis	augustus_masked-LADA0A-processed-gene-7.6
Kazachstania_martiniae	snap_masked-scf7180000030514-processed-gene-0.103
Lachancea_nothofagi	snap_masked-LANO0B-processed-gene-1.74
Kazachstania_kunashirensis	snap_masked-scf7180000008985-processed-gene-1.61
Lachancea_fantastica	snap_masked-LAFA0F-processed-gene-10.9
Tetrapisispora_iriomotensis	augustus_masked-flattened_line_57-processed-gene-0.130
Kazachstania_africana	augustus_masked-scaffold_2-processed-gene-13.205
Kluyveromyces_lactis	snap_masked-CR382122-processed-gene-3.49
Lachancea_meyersii	snap_masked-LAME0G-processed-gene-17.24
Kazachstania_siamensis	augustus_masked-NODE_61_length_63804_cov_14.6622_ID_121-processed-gene-0.80
Kluyveromyces_dobzhanskii	snap_masked-NODE_13_length_251415_cov_20.6991_ID_25-processed-gene-1.144
Eremothecium_cymbalariae	snap_masked-NC_016453.1-processed-gene-3.121
Lachancea_thermotolerans	snap_masked-CU928168-processed-gene-14.65
Kazachstania_taianensis	augustus_masked-NODE_5_length_452406_cov_49.1731_ID_9-processed-gene-3.120
Kazachstania_aerobia	augustus_masked-scf7180000020169-processed-gene-0.188
Kazachstania_unispora	snap_masked-NODE_47_length_78712_cov_50.1529_ID_93-processed-gene-0.49
Kazachstania_yakushimaensis	genemark-scf7180000044634-processed-gene-0.102
Kazachstania_spencerorum	snap_masked-scf7180000055600-processed-gene-0.48
Kazachstania_rosinii	snap_masked-scf7180000099615-processed-gene-0.31
Kazachstania_solicola	augustus_masked-scf7180000046332-processed-gene-1.206
Tetrapisispora_blattae	snap_masked-NC_020185-processed-gene-25.62
Tetrapisispora_namnaonensis	augustus_masked-NODE_17_length_366881_cov_78.9049_ID_33-processed-gene-2.114
Kluyveromyces_marxianus	snap_masked-BBIL01000008.1-processed-gene-11.40
Eremothecium_coryli	maker-AZAH01000009.1-snap-gene-0.44
Lachancea_nothofagi	snap_masked-LANO0B-processed-gene-3.66

>Nakaseomyces_bracarensis_g001614.m1 g001614.m1	g001614.m1	Nakaseomyces_bracarensis	1				Y1000_protID	Y1000_TreeID
>Nakaseomyces_castellii_g001576.m1 g001576.m1	g001576.m1	Nakaseomyces_castellii	1				g005344.m1	Saccharomyces_cerevisiae
>Nakaseomyces_glabratus_g000308.m1 g000308.m1	g000308.m1	Nakaseomyces_glabratus	1				g004292.m1	Saccharomyces_jurei
>Nakaseomyces_kungkrabaensis_g004198.m1 g004198.m1	g004198.m1	Nakaseomyces_kungkrabaensis	1				g005237.m1	Saccharomyces_paradoxus
>Nakaseomyces_nivariensis_g001471.m1 g001471.m1	g001471.m1	Nakaseomyces_nivariensis	1				g005184.m1	Saccharomyces_kudriavzevii
>Nakaseomyces_uthaithaninus_g005380.m1 g005380.m1	g005380.m1	Nakaseomyces_uthaithaninus	1				g005224.m1	Saccharomyces_arboricola
>Eremothecium_cymbalariae_g002832.m1 g002832.m1	g002832.m1	Eremothecium_cymbalariae	1				g005189.m1	Saccharomyces_mikatae
>Eremothecium_sinecaudum_g003584.m1 g003584.m1	g003584.m1	Eremothecium_sinecaudum	1				g002217.m1	Saccharomyces_uvarum
>Hagleromyces_aurorensis_g002965.m1 g002965.m1	g002965.m1	Hagleromyces_aurorensis	1				g005249.m1	Saccharomyces_eubayanus
>Kazachstania_africana_g003755.m1 g003755.m1	g003755.m1	Kazachstania_africana	1				g000308.m1	Nakaseomyces_glabratus
>Kazachstania_aquatica_g003032.m1 g003032.m1	g003032.m1	#N/A	0				g004198.m1	Nakaseomyces_kungkrabaensis
>Kazachstania_bromeliacearum_g000451.m1 g000451.m1	g000451.m1	Kazachstania_bromeliacearum	1				g001471.m1	Nakaseomyces_nivariensis
>Kazachstania_bulderi_g005958.m1 g005958.m1	g005958.m1	Kazachstania_bulderi	1				g005380.m1	Nakaseomyces_uthaithaninus
>Kazachstania_exigua_g000048.m1 g000048.m1	g000048.m1	#N/A	0				g004209.m1	Nakaseomyces_delphensis
>Kazachstania_exigua_g002519.m1 g002519.m1	g002519.m1	Kazachstania_exigua	1		1	g001614.m1	g001614.m1	Nakaseomyces_bracarensis
>Kazachstania_gamospora_g004167.m1 g004167.m1	g004167.m1	Kazachstania_gamospora	1				g004314.m1	Nakaseomyces_bacillisporus
>Kazachstania_hellenica_g003117.m1 g003117.m1	g003117.m1	Kazachstania_hellenica	1				g001576.m1	Nakaseomyces_castellii
>Kazachstania_humilis_NRRL_Y-7245_g000693.m1 g000693.m1	g000693.m1	#N/A	0				g005068.m1	Kazachstania_solicola
>Kazachstania_kunashirensis_g005050.m1 g005050.m1	g005050.m1	Kazachstania_kunashirensis	2				g002718.m1	Kazachstania_aerobia
>Kazachstania_lodderae_g003605.m1 g003605.m1	g003605.m1	Kazachstania_lodderae	1				g003649.m1	Kazachstania_unispora
>Kazachstania_martiniae_g004439.m1 g004439.m1	g004439.m1	Kazachstania_martiniae	1				g000451.m1	Kazachstania_bromeliacearum
>Kazachstania_naganishii_g001440.m1 g001440.m1	g001440.m1	Kazachstania_naganishii	1				g005958.m1	Kazachstania_bulderi
>Kazachstania_piceae_g005225.m1 g005225.m1	g005225.m1	Kazachstania_piceae	1				g002519.m1	Kazachstania_exigua
>Kazachstania_pseudohumilis_g002365.m1 g002365.m1	g002365.m1	#N/A	0				g004167.m1	Kazachstania_gamospora
>Kazachstania_rosinii_g001213.m1 g001213.m1	g001213.m1	#N/A	0				g003117.m1	Kazachstania_hellenica
>Kazachstania_saulgeensis_g000550.m1 g000550.m1	g000550.m1	Kazachstania_saulgeensis	1				g005050.m1	Kazachstania_kunashirensis
>Kazachstania_servazzii_g000773.m1 g000773.m1	g000773.m1	Kazachstania_servazzii	1				g003605.m1	Kazachstania_lodderae
>Kazachstania_siamensis_g004344.m1 g004344.m1	g004344.m1	Kazachstania_siamensis	1				g005225.m1	Kazachstania_piceae
>Kazachstania_sinensis_g005599.m1 g005599.m1	g005599.m1	Kazachstania_sinensis	1				g000550.m1	Kazachstania_saulgeensis
>Kazachstania_sp._UFMG-CM-Y273_g000466.m1 g000466.m1	g000466.m1	#N/A	0				g000773.m1	Kazachstania_servazzii
>Kazachstania_taianensis_g004007.m1 g004007.m1	g004007.m1	Kazachstania_taianensis	1				g005599.m1	Kazachstania_sinensis
>Kazachstania_turicensis_g004713.m1 g004713.m1	g004713.m1	Kazachstania_turicensis	1				g001209.m1	Kazachstania_yasuniensis
>Kazachstania_viticola_g001183.m1 g001183.m1	g001183.m1	Kazachstania_viticola	1				g001263.m1	Kazachstania_barnettii
>Kazachstania_yasuniensis_g001209.m1 g001209.m1	g001209.m1	Kazachstania_yasuniensis	1				g002942.m1	Kazachstania_psychrophila
>Kluyveromyces_aestuarii_ATCC_18862_g000823.m1 g000823.m1	g000823.m1	Kluyveromyces_aestuarii_ATCC_18862	1				g001801.m1	Kazachstania_jinghongensis
>Kluyveromyces_aestuarii_NRRL_YB-4510_g003160.m1 g003160.m1	g003160.m1	#N/A	0				g004344.m1	Kazachstania_siamensis
>Kluyveromyces_dobzhanskii_g001256.m1 g001256.m1	g001256.m1	Kluyveromyces_dobzhanskii	1				g004007.m1	Kazachstania_taianensis
>Kluyveromyces_lactis_CBS_2359_g000677.m1 g000677.m1	g000677.m1	Kluyveromyces_lactis_CBS_2359	1				g001440.m1	Kazachstania_naganishii
>Kluyveromyces_lactis_var._drosophilarum_NRRL_Y-8278_g001346.m1 g001346.m1	g001346.m1	#N/A	0				g004439.m1	Kazachstania_martiniae
>Kluyveromyces_lactis_var._lactis_NRRL_Y-8279_g002882.m1 g002882.m1	g002882.m1	#N/A	0				g004713.m1	Kazachstania_turicensis
>Kluyveromyces_nonfermentans_g001343.m1 g001343.m1	g001343.m1	Kluyveromyces_nonfermentans	1				g005050.m1	Kazachstania_kunashirensis
>Kluyveromyces_siamensis_g003460.m1 g003460.m1	g003460.m1	Kluyveromyces_siamensis	1				g002874.m1	Kazachstania_spencerorum
>Kluyveromyces_sp._yHMH660_g005129.m1 g005129.m1	g005129.m1	#N/A	0				g003755.m1	Kazachstania_africana
>Kluyveromyces_starmeri_g003161.m1 g003161.m1	g003161.m1	Kluyveromyces_starmeri	1				g001183.m1	Kazachstania_viticola
>Kluyveromyces_wickerhamii_g000532.m1 g000532.m1	g000532.m1	Kluyveromyces_wickerhamii	1				g002752.m1	Naumovozyma_dairenensis
>Lachancea_cidri_NRRL_Y-12635_g002916.m1 g002916.m1	g002916.m1	#N/A	0				g001098.m1	Naumovozyma_castellii
>Lachancea_cidri_NRRL_Y-12634_g003378.m1 g003378.m1	g003378.m1	#N/A	0				g000794.m1	Naumovozyma_baii
>Lachancea_dasiensis_g003259.m1 g003259.m1	g003259.m1	Lachancea_dasiensis	1				g003416.m1	Tetrapisispora_iriomotensis
>Lachancea_fantastica_nom._nud._g003435.m1 g003435.m1	g003435.m1	Lachancea_fantastica_nom._nud.	1				g001993.m1	Tetrapisispora_namnaoensis
>Lachancea_fermentati_g004518.m1 g004518.m1	g004518.m1	Lachancea_fermentati	1				g003018.m1	Tetrapisispora_blattae
>Lachancea_kluyveri_g001117.m1 g001117.m1	g001117.m1	Lachancea_kluyveri	1				g005365.m1	Vanderwaltozyma_polyspora
>Lachancea_meyersii_g001214.m1 g001214.m1	g001214.m1	Lachancea_meyersii	1				g001449.m1	Vanderwaltozyma_yarrowii
>Lachancea_mirantina_g001548.m1 g001548.m1	g001548.m1	Lachancea_mirantina	1				g003717.m1	Vanderwaltozyma_tropicalis
>Lachancea_nothofagi_g001406.m1 g001406.m1	g001406.m1	Lachancea_nothofagi	1				g005143.m1	Vanderwaltozyma_verrucispora
>Lachancea_quebecensis_g002616.m1 g002616.m1	g002616.m1	Lachancea_quebecensis	1				g004938.m1	Torulaspora_pretoriensis
>Lachancea_sp._yHQL494_g002832.m1 g002832.m1	g002832.m1	Eremothecium_cymbalariae	1				g002639.m1	Torulaspora_microellipsoides
>Lachancea_thermotolerans_g003213.m1 g003213.m1	g003213.m1	Lachancea_thermotolerans	1				g004752.m1	Torulaspora_franciscae
>Lachancea_waltii_g003199.m1 g003199.m1	g003199.m1	Lachancea_waltii	1				g001776.m1	Torulaspora_delbrueckii
>Kazachstania_barnettii_g001263.m1 g001263.m1	g001263.m1	Kazachstania_barnettii	1				g001826.m1	Torulaspora_globosa
>Torulaspora_franciscae_g004752.m1 g004752.m1	g004752.m1	Torulaspora_franciscae	1				g003867.m1	Torulaspora_maleeae
>Lachancea_lanzarotensis_g004947.m1 g004947.m1	g004947.m1	Lachancea_lanzarotensis	1				g005101.m1	Zygosaccharomyces_bisporus
>Kazachstania_psychrophila_g002942.m1 g002942.m1	g002942.m1	Kazachstania_psychrophila	1				g005022.m1	Zygosaccharomyces_gambellarensis
>Vanderwaltozyma_yarrowii_g001449.m1 g001449.m1	g001449.m1	Vanderwaltozyma_yarrowii	1				g001048.m1	Zygosaccharomyces_bailii
>Tetrapisispora_iriomotensis_g003416.m1 g003416.m1	g003416.m1	Tetrapisispora_iriomotensis	1				g000063.m1	Zygosaccharomyces_kombuchaensis
>Torulaspora_pretoriensis_g004938.m1 g004938.m1	g004938.m1	Torulaspora_pretoriensis	1				g004388.m1	Zygosaccharomyces_lentus
>Torulaspora_microellipsoides_g002639.m1 g002639.m1	g002639.m1	Torulaspora_microellipsoides	1				g002135.m1	Zygosaccharomyces_mellis
>Kazachstania_jinghongensis_g001801.m1 g001801.m1	g001801.m1	Kazachstania_jinghongensis	1				g004410.m1	Zygosaccharomyces_pseudorouxii
>Vanderwaltozyma_verrucispora_g005143.m1 g005143.m1	g005143.m1	Vanderwaltozyma_verrucispora	1				g000372.m1	Zygosaccharomyces_siamensis
>Nakaseomyces_bacillisporus_g004314.m1 g004314.m1	g004314.m1	Nakaseomyces_bacillisporus	1				g004327.m1	Zygotorulaspora_florentina
>Nakaseomyces_delphensis_g004209.m1 g004209.m1	g004209.m1	Nakaseomyces_delphensis	1				g002003.m1	Zygotorulaspora_mrakii
>Nakaseomyces_sp._yHDO568_g004296.m1 g004296.m1	g004296.m1	#N/A	0				g002973.m1	Zygotorulaspora_chibaensis
>Naumovozyma_baii_g000794.m1 g000794.m1	g000794.m1	Naumovozyma_baii	1				g002121.m1	Zygotorulaspora_danielsina
>Naumovozyma_castellii_g001098.m1 g001098.m1	g001098.m1	Naumovozyma_castellii	1				g002003.m1	Zygotorulaspora_mrakii
>Naumovozyma_dairenensis_g002752.m1 g002752.m1	g002752.m1	Naumovozyma_dairenensis	1				g002965.m1	Hagleromyces_aurorensis

>Saccharomyces_arboricola_g005224.m1 g005224.m1	g005224.m1	Saccharomyces_arboricola	1				*g001117.m1*	*Lachancea_kluyveri*
>Saccharomyces_cerevisiae_g005344.m1 g005344.m1	g005344.m1	Saccharomyces_cerevisiae	1				*g001548.m1*	*Lachancea_mirantina*
>Saccharomyces_eubayanus_g005249.m1 g005249.m1	g005249.m1	Saccharomyces_eubayanus	1				*g001406.m1*	*Lachancea_nothofagi*
>Saccharomyces_jurei_g004292.m1 g004292.m1	g004292.m1	Saccharomyces_jurei	1				*g003435.m1*	Lachancea_fantastica_nom._nud.
>Saccharomyces_kudriavzevii_g005184.m1 g005184.m1	g005184.m1	Saccharomyces_kudriavzevii	1				*g004947.m1*	*Lachancea_lanzarotensis*
>Saccharomyces_mikatae_g005189.m1 g005189.m1	g005189.m1	Saccharomyces_mikatae	1				*g003199.m1*	*Lachancea_waltii*
>Saccharomyces_paradoxus_g005237.m1 g005237.m1	g005237.m1	Saccharomyces_paradoxus	1				g002616.m1	*Lachancea_quebecensis*
>Saccharomyces_uvarum_g002217.m1 g002217.m1	g002217.m1	Saccharomyces_uvarum	1				*g003213.m1*	*Lachancea_thermotolerans*
>Torulaspora_delbrueckii_g001776.m1 g001776.m1	g001776.m1	Torulaspora_delbrueckii	1				*g001214.m1*	*Lachancea_meyersii*
>Torulaspora_globosa_g001826.m1 g001826.m1	g001826.m1	Torulaspora_globosa	1				*g003259.m1*	*Lachancea_dasiensis*
>Torulaspora_maleeae_g003867.m1 g003867.m1	g003867.m1	Torulaspora_maleeae	1				g004518.m1	*Lachancea_fermentati*
>Torulaspora_sp._yHMJ407_g002906.m1 g002906.m1	g002906.m1	#N/A	0				*NA*	Eremothecium_gossypii
>Vanderwaltozyma_polyspora_g005365.m1 g005365.m1	g005365.m1	Vanderwaltozyma_polyspora	1				*g002832.m1*	*Eremothecium_cymbalariae*
>Vanderwaltozyma_tropicalis_g003717.m1 g003717.m1	g003717.m1	Vanderwaltozyma_tropicalis	2				*g004362.m1*	*Eremothecium_coryli*
>Zygosaccharomyces_bailii_g001048.m1 g001048.m1	g001048.m1	Zygosaccharomyces_bailii	1				*g003584.m1*	*Eremothecium_sinecaudum*
>Zygosaccharomyces_bisporus_g005101.m1 g005101.m1	g005101.m1	Zygosaccharomyces_bisporus	1				g000677.m1	Kluyveromyces_lactis_CBS_2359
>Zygosaccharomyces_gambellarensis_g005022.m1 g005022.m1	g005022.m1	Zygosaccharomyces_gambellarensis	1				*g001256.m1*	*Kluyveromyces_dobzhanskii*
>Zygosaccharomyces_kombuchaensis_g000063.m1 g000063.m1	g000063.m1	Zygosaccharomyces_kombuchaensis	1				g000823.m1	Kluyveromyces_aestuarii_ATCC_18862
>Zygosaccharomyces_lentus_g004388.m1 g004388.m1	g004388.m1	Zygosaccharomyces_lentus	1				*g001343.m1*	*Kluyveromyces_nonfermentans*
>Zygosaccharomyces_mellis_g002135.m1 g002135.m1	g002135.m1	Zygosaccharomyces_mellis	1				*g003460.m1*	*Kluyveromyces_siamensis*
>Zygosaccharomyces_parabailii_g003412.m1 g003412.m1	g003412.m1	#N/A	0				*g003161.m1*	*Kluyveromyces_starmeri*
>Zygosaccharomyces_pseudobailii_g001287.m1 g001287.m1	g001287.m1	#N/A	0				*g000532.m1*	*Kluyveromyces_wickerhamii*
>Zygosaccharomyces_pseudobailii_g002625.m1 g002625.m1	g002625.m1	#N/A	0				*g000873.m1*	Hanseniaspora_uvarum_DSMZ_2768
>Zygosaccharomyces_pseudobailii_g003112.m1 g003112.m1	g003112.m1	#N/A	0				*g000765.m1*	Hanseniaspora_pseudoguilliermondii
>Zygosaccharomyces_pseudorouxii_g004410.m1 g004410.m1	g004410.m1	Zygosaccharomyces_pseudorouxii	1				*g003387.m1*	*Hanseniaspora_clermontiae*
>Zygosaccharomyces_rouxii_g001572.m1 g001572.m1	g001572.m1	#N/A	0				g000788.m1	*Hanseniaspora_valbyensis*
>Zygosaccharomyces_sapae_g000017.m1 g000017.m1	g000017.m1	#N/A	0				g000163.m1	Hanseniaspora_vineae
>Zygosaccharomyces_sapae_g006118.m1 g006118.m1	g006118.m1	#N/A	0				*g005674.m1*	*Cyberlindnera_suaveolens*
>Zygosaccharomyces_siamensis_g000372.m1 g000372.m1	g000372.m1	Zygosaccharomyces_siamensis	1				*g001009.m1*	*Cyberlindnera_saturnus*
>Zygotorulaspora_chibaensis_g002973.m1 g002973.m1	g002973.m1	Zygotorulaspora_chibaensis	1				*g002417.m1*	*Cyberlindnera_misumaiensis*
>Zygotorulaspora_danielsina_g002121.m1 g002121.m1	g002121.m1	Zygotorulaspora_danielsina	1				*g002673.m1*	*Cyberlindnera_jadinii*
>Zygotorulaspora_florentina_g004327.m1 g004327.m1	g004327.m1	Zygotorulaspora_florentina	1				*g005778.m1*	*Cyberlindnera_fabianii*
>Zygotorulaspora_mrakii_g002003.m1 g002003.m1	g002003.m1	Zygotorulaspora_mrakii	2				*g003717.m1*	*Cyberlindnera_maclurae*
>Zygotorulaspora_sp._yHDO592_g004529.m1 g004529.m1	g004529.m1	#N/A	0				*g001244.m1*	Wickerhamomyces_alni
							g004559.m1	*Wickerhamomyces_canadensis*
							g005482.m1,g003054.m1	*Wickerhamomyces_ciferrii*
							g001027.m1	*Wickerhamomyces_chambardii*
							g002138.m1	*Wickerhamomyces_hampshirensis*
							g002404.m1	*Wickerhamomyces_mucosus*
							g002584.m1	Wickerhamomyces_pijperi
							g002262.m1	Komagataella_phaffii_GS115
							g004657.m1	*Komagataella_populi*
							g002679.m1	Komagataella_pastoris_ATCC_28485
							g004231.m1	*Komagataella_kurtzmanii*
							g004487.m1	Komagataella_mondaviorum
							g004700.m1	*Kuraishia_floccosa*
							NA	Ashbya_aceri
							g001657.m1	Wickerhamomyces_anomalus

Species	gID	evalue	score	fastaID	countInTableS1		Y1000_protID_TableS1	count_in_hmm_results	Present_in_hmm_results
Nakaseomyces_bracarensis	g001614.m1	1.00E-23	82.7	>Nakaseomyces_bracarensis_g001614.m1 g001614.m1	1		g005344.m1	1	1
Nakaseomyces_castellii	g001576.m1	8.20E-21	73.1	>Nakaseomyces_castellii_g001576.m1 g001576.m1	1		g004292.m1	1	1
Nakaseomyces_glabratus	g000308.m1	2.00E-21	75.2	>Nakaseomyces_glabratus_g000308.m1 g000308.m1	1		g005237.m1	1	1
Nakaseomyces_kungkrabaensis	g004198.m1	6.30E-23	80.1	>Nakaseomyces_kungkrabaensis_g004198.m1 g004198.m1	1		g005184.m1	1	1
Nakaseomyces_nivariensis	g001471.m1	8.60E-23	79.6	>Nakaseomyces_nivariensis_g001471.m1 g001471.m1	1		g005224.m1	1	1
Nakaseomyces_uthaithaninus	g005380.m1	1.30E-22	79.1	>Nakaseomyces_uthaithaninus_g005380.m1 g005380.m1	1		g005189.m1	1	1
Eremothecium_cymbalariae	g002832.m1	5.50E-15	53.8	>Eremothecium_cymbalariae_g002832.m1 g002832.m1	1		g002217.m1	1	1
Eremothecium_sinecaudum	g003584.m1	8.20E-17	59.8	>Eremothecium_sinecaudum_g003584.m1 g003584.m1	1		g005249.m1	1	1
Hagleromyces_aurorensis	g002965.m1	4.10E-38	129.9	>Hagleromyces_aurorensis_g002965.m1 g002965.m1	1		g000308.m1	1	1
Kazachstania_africana	g003755.m1	1.40E-17	62.6	>Kazachstania_africana_g003755.m1 g003755.m1	1		g004198.m1	1	1
Kazachstania_aquatica	g003032.m1	5.10E-20	70.7	>Kazachstania_aquatica_g003032.m1 g003032.m1	1		g001471.m1	1	1
Kazachstania_bromeliacearum	g000451.m1	6.40E-21	73.6	>Kazachstania_bromeliacearum_g000451.m1 g000451.m1	1		g005380.m1	1	1
Kazachstania_bulderi	g005958.m1	2.50E-16	58.6	>Kazachstania_bulderi_g005958.m1 g005958.m1	1		g004209.m1	1	1
Kazachstania_exigua	g002519.m1	5.00E-17	61.7	>Kazachstania_exigua_g002519.m1 g002519.m1	1		g001614.m1	1	1
Kazachstania_exigua	g000048.m1	1.50E-14	53.6	>Kazachstania_exigua_g000048.m1 g000048.m1	0		g004314.m1	1	1
Kazachstania_gamospora	g004167.m1	3.00E-17	61.5	>Kazachstania_gamospora_g004167.m1 g004167.m1	1		g001576.m1	1	1
Kazachstania_hellenica	g003117.m1	7.90E-20	69.9	>Kazachstania_hellenica_g003117.m1 g003117.m1	1		g005068.m1	0	0
Kazachstania_humilis_NRRL_Y-7245	g000693.m1	2.10E-17	62.3	>Kazachstania_humilis_NRRL_Y-7245_g000693.m1 g000693.m1	0		g002718.m1	0	0
Kazachstania_kunashirensis	g005050.m1	9.30E-19	66.5	>Kazachstania_kunashirensis_g005050.m1 g005050.m1	2		g003649.m1	0	0
Kazachstania_lodderae	g003605.m1	4.30E-19	67.6	>Kazachstania_lodderae_g003605.m1 g003605.m1	1		g000451.m1	1	1
Kazachstania_martiniae	g004439.m1	5.20E-18	64.1	>Kazachstania_martiniae_g004439.m1 g004439.m1	1		g005958.m1	1	1
Kazachstania_naganishii	g001440.m1	1.20E-19	69.3	>Kazachstania_naganishii_g001440.m1 g001440.m1	1		g002519.m1	1	1
Kazachstania_piceae	g005225.m1	5.50E-18	64	>Kazachstania_piceae_g005225.m1 g005225.m1	1		g004167.m1	1	1
Kazachstania_pseudohumilis	g002365.m1	1.00E-16	59.9	>Kazachstania_pseudohumilis_g002365.m1 g002365.m1	0		g003117.m1	1	1
Kazachstania_rosinii	g001213.m1	3.70E-20	71.1	>Kazachstania_rosinii_g001213.m1 g001213.m1	0		g003032.m1	1	1
Kazachstania_saulgeensis	g000550.m1	2.80E-18	64.9	>Kazachstania_saulgeensis_g000550.m1 g000550.m1	1		g005050.m1	1	1
Kazachstania_servazzii	g000773.m1	6.60E-17	60.4	>Kazachstania_servazzii_g000773.m1 g000773.m1	1		g003605.m1	1	1
Kazachstania_siamensis	g004344.m1	4.50E-17	60.9	>Kazachstania_siamensis_g004344.m1 g004344.m1	1		g005225.m1	1	1
Kazachstania_sinensis	g005599.m1	3.60E-21	74.4	>Kazachstania_sinensis_g005599.m1 g005599.m1	1		g000550.m1	1	1
Kazachstania_sp._UFMG-CM-Y273	g000466.m1	2.20E-18	65.2	>Kazachstania_sp._UFMG-CM-Y273_g000466.m1 g000466.m1	0		g000773.m1	1	1
Kazachstania_taianensis	g004007.m1	3.30E-14	51.5	>Kazachstania_taianensis_g004007.m1 g004007.m1	1		g005599.m1	1	1
Kazachstania_turicensis	g004713.m1	5.80E-20	70.4	>Kazachstania_turicensis_g004713.m1 g004713.m1	1		g001209.m1	1	1
Kazachstania_viticola	g001183.m1	3.00E-34	117.3	>Kazachstania_viticola_g001183.m1 g001183.m1	1		g001263.m1	1	1
Kazachstania_yasuniensis	g001209.m1	2.90E-15	55	>Kazachstania_yasuniensis_g001209.m1 g001209.m1	1		g002942.m1	1	1
Kluyveromyces_aestuarii_ATCC_18862	g000823.m1	1.00E-24	85.9	>Kluyveromyces_aestuarii_ATCC_18862_g000823.m1 g000823.m1	1		g001801.m1	1	1
Kluyveromyces_aestuarii_NRRL_YB-4510	g003160.m1	1.00E-24	85.9	>Kluyveromyces_aestuarii_NRRL_YB-4510_g003160.m1 g003160.m1	0		g004344.m1	1	1
Kluyveromyces_dobzhanskii	g001256.m1	2.00E-15	55.4	>Kluyveromyces_dobzhanskii_g001256.m1 g001256.m1	1		g004007.m1	1	1
Kluyveromyces_lactis_CBS_2359	g000677.m1	2.70E-17	61.5	>Kluyveromyces_lactis_CBS_2359_g000677.m1 g000677.m1	1		g001440.m1	1	1
Kluyveromyces_lactis_var._drosophilarum_NRRL_Y-8278	g001346.m1	6.30E-18	63.6	>Kluyveromyces_lactis_var._drosophilarum_NRRL_Y-8278_g001346.m1 g001346.m1	0		g004439.m1	1	1
Kluyveromyces_lactis_var._lactis_NRRL_Y-8279	g002882.m1	2.70E-17	61.5	>Kluyveromyces_lactis_var._lactis_NRRL_Y-8279_g002882.m1 g002882.m1	0		g004713.m1	1	1
Kluyveromyces_nonfermentans	g001343.m1	1.10E-21	75.9	>Kluyveromyces_nonfermentans_g001343.m1 g001343.m1	1		g005050.m1	1	1
Kluyveromyces_siamensis	g003460.m1	1.50E-25	88.7	>Kluyveromyces_siamensis_g003460.m1 g003460.m1	1		g002874.m1	0	0
Kluyveromyces_sp._yHMH660	g005129.m1	8.70E-14	50.1	>Kluyveromyces_sp._yHMH660_g005129.m1 g005129.m1	0		g003755.m1	1	1
Kluyveromyces_starmeri	g003161.m1	5.50E-15	53.9	>Kluyveromyces_starmeri_g003161.m1 g003161.m1	1		g001183.m1	1	1
Kluyveromyces_wickerhamii	g000532.m1	1.90E-18	65.3	>Kluyveromyces_wickerhamii_g000532.m1 g000532.m1	1		g002752.m1	1	1
Lachancea_cidri_NRRL_Y-12635	g002916.m1	6.50E-29	99.7	>Lachancea_cidri_NRRL_Y-12635_g002916.m1 g002916.m1	0		g001098.m1	1	1
Lachancea_cidri_NRRL_Y-12634	g003378.m1	6.60E-29	99.7	>Lachancea_cidri_NRRL_Y-12634_g003378.m1 g003378.m1	0		g000794.m1	1	1
Lachancea_dasiensis	g003259.m1	7.30E-19	66.7	>Lachancea_dasiensis_g003259.m1 g003259.m1	1		g003416.m1	1	1
Lachancea_fantastica_nom._nud.	g003435.m1	9.60E-19	66.3	>Lachancea_fantastica_nom._nud._g003435.m1 g003435.m1	1		g001993.m1	0	0
Lachancea_fermentati	g004518.m1	2.30E-29	101.2	>Lachancea_fermentati_g004518.m1 g004518.m1	1		g003018.m1	0	0
Lachancea_kluyveri	g001117.m1	5.90E-82	274.1	>Lachancea_kluyveri_g001117.m1 g001117.m1	1		g005365.m1	1	1
Lachancea_meyersii	g001214.m1	1.20E-18	66	>Lachancea_meyersii_g001214.m1 g001214.m1	1		g001449.m1	1	1
Lachancea_mirantina	g001548.m1	3.50E-23	80.9	>Lachancea_mirantina_g001548.m1 g001548.m1	1		g003717.m1	1	1
Lachancea_nothofagi	g001406.m1	5.90E-24	83.4	>Lachancea_nothofagi_g001406.m1 g001406.m1	1		g005143.m1	1	1
Lachancea_quebecensis	g002616.m1	4.00E-28	97.1	>Lachancea_quebecensis_g002616.m1 g002616.m1	1		g004938.m1	1	1
Lachancea_sp._yHQL494	g002832.m1	5.20E-29	100	>Lachancea_sp._yHQL494_g002832.m1 g002832.m1	1		g002639.m1	1	1
Lachancea_thermotolerans	g003213.m1	3.60E-28	97.3	>Lachancea_thermotolerans_g003213.m1 g003213.m1	1		g004752.m1	1	1
Lachancea_waltii	g003199.m1	5.20E-25	86.9	>Lachancea_waltii_g003199.m1 g003199.m1	1		g001776.m1	1	1
Kazachstania_barnettii	g001263.m1	3.00E-18	64.8	>Kazachstania_barnettii_g001263.m1 g001263.m1	1		g001826.m1	1	1
Torulaspora_franciscae	g004752.m1	1.30E-107	358.3	>Torulaspora_franciscae_g004752.m1 g004752.m1	1		g003867.m1	1	1
Lachancea_lanzarotensis	g004947.m1	1.30E-19	69.2	>Lachancea_lanzarotensis_g004947.m1 g004947.m1	1		g005101.m1	1	1
Kazachstania_psychrophila	g002942.m1	5.50E-19	67.2	>Kazachstania_psychrophila_g002942.m1 g002942.m1	1		g005022.m1	1	1
Vanderwaltozyma_yarrowii	g001449.m1	1.90E-27	95	>Vanderwaltozyma_yarrowii_g001449.m1 g001449.m1	1		g001048.m1	1	1
Tetrapisispora_iriomotensis	g003416.m1	2.40E-18	65	>Tetrapisispora_iriomotensis_g003416.m1 g003416.m1	1		g000063.m1	1	1
Torulaspora_pretoriensis	g004938.m1	6.40E-105	349.4	>Torulaspora_pretoriensis_g004938.m1 g004938.m1	1		g004388.m1	1	1
Torulaspora_microellipsoides	g002639.m1	1.00E-101	339	>Torulaspora_microellipsoides_g002639.m1 g002639.m1	1		g002135.m1	1	1
Kazachstania_jinghongensis	g001801.m1	1.20E-15	56.2	>Kazachstania_jinghongensis_g001801.m1 g001801.m1	1		g004410.m1	1	1
Vanderwaltozyma_verrucispora	g005143.m1	2.30E-33	114.5	>Vanderwaltozyma_verrucispora_g005143.m1 g005143.m1	1		g000372.m1	1	1
Nakaseomyces_bacillisporus	g004314.m1	1.50E-24	85.4	>Nakaseomyces_bacillisporus_g004314.m1 g004314.m1	1		g004327.m1	1	1
Nakaseomyces_delphensis	g004209.m1	1.70E-27	95.1	>Nakaseomyces_delphensis_g004209.m1 g004209.m1	1		g002003.m1	1	1

Species	gID	evalue	score	fastaID	countInTableS1		Y1000_protID_TableS1	count_in_hmm_results	Present_in_hmm_results
Nakaseomyces_sp._yHDO568	g004296.m1	1.30E-24	85.7	>Nakaseomyces_sp._yHDO568_g004296.m1 g004296.m1	0		g002973.m1	1	1
Naumovozyma_baii	g000794.m1	3.60E-30	104	>Naumovozyma_baii_g000794.m1 g000794.m1	1		g002121.m1	1	1
Naumovozyma_castellii	g001098.m1	5.40E-47	159.3	>Naumovozyma_castellii_g001098.m1 g001098.m1	1		g002003.m1	1	1
Naumovozyma_dairenensis	g002752.m1	1.70E-51	174.1	>Naumovozyma_dairenensis_g002752.m1 g002752.m1	1		g002965.m1	1	1
Saccharomyces_arboricola	g005224.m1	1.60E-116	387.7	>Saccharomyces_arboricola_g005224.m1 g005224.m1	1		g001117.m1	1	1
Saccharomyces_cerevisiae	g005344.m1	2.70E-131	436.2	>Saccharomyces_cerevisiae_g005344.m1 g005344.m1	1		g001548.m1	1	1
Saccharomyces_eubayanus	g005249.m1	8.10E-123	408.4	>Saccharomyces_eubayanus_g005249.m1 g005249.m1	1		g001406.m1	1	1
Saccharomyces_jurei	g004292.m1	3.90E-128	425.9	>Saccharomyces_jurei_g004292.m1 g004292.m1	1		g003435.m1	1	1
Saccharomyces_kudriavzevii	g005184.m1	1.60E-126	420.5	>Saccharomyces_kudriavzevii_g005184.m1 g005184.m1	1		g004947.m1	1	1
Saccharomyces_mikatae	g005189.m1	4.50E-133	442.1	>Saccharomyces_mikatae_g005189.m1 g005189.m1	1		g003199.m1	1	1
Saccharomyces_paradoxus	g005237.m1	6.30E-133	441.6	>Saccharomyces_paradoxus_g005237.m1 g005237.m1	1		g002616.m1	1	1
Saccharomyces_uvarum	g002217.m1	3.10E-128	426.2	>Saccharomyces_uvarum_g002217.m1 g002217.m1	1		g003213.m1	1	1
Torulaspora_delbrueckii	g001776.m1	2.40E-108	360.7	>Torulaspora_delbrueckii_g001776.m1 g001776.m1	1		g001214.m1	1	1
Torulaspora_globosa	g001826.m1	1.20E-71	240.1	>Torulaspora_globosa_g001826.m1 g001826.m1	1		g003259.m1	1	1
Torulaspora_maleeae	g003867.m1	9.50E-89	296.3	>Torulaspora_maleeae_g003867.m1 g003867.m1	1		g004518.m1	1	1
Torulaspora_sp._yHMJ407	g002906.m1	9.60E-81	270	>Torulaspora_sp._yHMJ407_g002906.m1 g002906.m1	0		NA	0	0
Vanderwaltozyma_polyspora	g005365.m1	3.40E-25	87.6	>Vanderwaltozyma_polyspora_g005365.m1 g005365.m1	1		g002832.m1	2	1
Vanderwaltozyma_tropicalis	g003717.m1	2.40E-26	91.4	>Vanderwaltozyma_tropicalis_g003717.m1 g003717.m1	2		g004362.m1	0	0
Zygosaccharomyces_bailii	g001048.m1	1.20E-100	335.5	>Zygosaccharomyces_bailii_g001048.m1 g001048.m1	1		g003584.m1	1	1
Zygosaccharomyces_bisporus	g005101.m1	4.00E-95	317.3	>Zygosaccharomyces_bisporus_g005101.m1 g005101.m1	1		g000677.m1	1	1
Zygosaccharomyces_gambellarensis	g005022.m1	3.20E-88	294.6	>Zygosaccharomyces_gambellarensis_g005022.m1 g005022.m1	1		g001256.m1	1	1
Zygosaccharomyces_kombuchaensis	g000063.m1	1.00E-95	319.2	>Zygosaccharomyces_kombuchaensis_g000063.m1 g000063.m1	1		g000823.m1	1	1
Zygosaccharomyces_lentus	g004388.m1	8.20E-88	293.3	>Zygosaccharomyces_lentus_g004388.m1 g004388.m1	1		g001343.m1	1	1
Zygosaccharomyces_mellis	g002135.m1	4.30E-86	287.6	>Zygosaccharomyces_mellis_g002135.m1 g002135.m1	1		g003460.m1	1	1
Zygosaccharomyces_parabailii	g003412.m1	1.20E-100	335.5	>Zygosaccharomyces_parabailii_g003412.m1 g003412.m1	0		g003161.m1	1	1
Zygosaccharomyces_pseudobailii	g001287.m1	2.00E-100	335.5	>Zygosaccharomyces_pseudobailii_g001287.m1 g001287.m1	0		g000532.m1	1	1
Zygosaccharomyces_pseudobailii	g002625.m1	2.90E-67	226.5	>Zygosaccharomyces_pseudobailii_g002625.m1 g002625.m1	0		g000873.m1	0	0
Zygosaccharomyces_pseudobailii	g003112.m1	7.60E-58	195.6	>Zygosaccharomyces_pseudobailii_g003112.m1 g003112.m1	0		g000765.m1	0	0
Zygosaccharomyces_pseudorouxii	g004410.m1	6.80E-94	313.2	>Zygosaccharomyces_pseudorouxii_g004410.m1 g004410.m1	1		g003387.m1	0	0
Zygosaccharomyces_rouxii	g001572.m1	9.90E-94	312.7	>Zygosaccharomyces_rouxii_g001572.m1 g001572.m1	0		g000788.m1	0	0
Zygosaccharomyces_sapae	g000017.m1	1.40E-93	312.7	>Zygosaccharomyces_sapae_g000017.m1 g000017.m1	0		g000163.m1	0	0
Zygosaccharomyces_sapae	g006118.m1	2.00E-89	299	>Zygosaccharomyces_sapae_g006118.m1 g006118.m1	0		g005674.m1	0	0
Zygosaccharomyces_siamensis	g000372.m1	2.40E-83	278.5	>Zygosaccharomyces_siamensis_g000372.m1 g000372.m1	1		g001009.m1	0	0
Zygotorulaspora_chibaensis	g002973.m1	5.30E-86	287.3	>Zygotorulaspora_chibaensis_g002973.m1 g002973.m1	1		g002417.m1	0	0
Zygotorulaspora_danielsina	g002121.m1	3.90E-77	258.2	>Zygotorulaspora_danielsina_g002121.m1 g002121.m1	1		g002673.m1	0	0
Zygotorulaspora_florentina	g004327.m1	2.20E-100	334.5	>Zygotorulaspora_florentina_g004327.m1 g004327.m1	1		g005778.m1	0	0
Zygotorulaspora_mrakii	g002003.m1	4.20E-94	313.9	>Zygotorulaspora_mrakii_g002003.m1 g002003.m1	2		g003717.m1	1	1
Zygotorulaspora_sp._yHDO592	g004529.m1	3.50E-60	202.5	>Zygotorulaspora_sp._yHDO592_g004529.m1 g004529.m1	0		g001244.m1	0	0
							g004559.m1	0	0
							g005482.m1,g003054.m1	0	0
							g001027.m1	0	0
							g002138.m1	0	0
							g002404.m1	0	0
							g002584.m1	0	0
							g002262.m1	0	0
							g004657.m1	0	0
							g002679.m1	0	0
							g004231.m1	0	0
							g004487.m1	0	0
							g004700.m1	0	0
							NA	0	0
							g001657.m1	0	0
